# From Rough Lapping to Fine Lapping: A Systematic Study on Tool-Material Compatibility and Process Parameter Optimization for Polycrystalline Diamond

**DOI:** 10.3390/mi17080895

**Published:** 2026-07-26

**Authors:** Yicun Zhu, Bingsan Chen, Yongchao Xu, Hongping Liao, Chunyu Li, Shusheng Chen

**Affiliations:** 1 Castech Inc., Fuzhou 350003, China; zhuyicun@castech.com (Y.Z.); liaohongping@castech.com (H.L.); fjqzdh8888@163.com (S.C.); 2 Fujian Key Laboratory of Intelligent Machining Technology and Equipment, Fujian University of Technology, Fuzhou 350118, China; 19862091@fjut.edu.cn (Y.X.); 19801546@fjut.edu.cn (C.L.)

**Keywords:** polycrystalline diamond (PCD), diamond abrasive disc, surface roughness, mechanical lapping

## Abstract

Polycrystalline diamond (PCD) has broad application prospects in semiconductors, optical windows and other advanced fields. Nevertheless, its ultrahigh hardness and chemical inertness pose significant challenges for achieving high-quality surface planarization. Although conventional mechanical lapping is widely adopted, it still suffers from poor surface quality and a lack of theoretical guidance for process parameter selection. This study presents a systematic experimental and simulation investigation on both rough and fine lapping of PCD, focusing on tool selection and process optimization. In the rough lapping stage, three types of fixed diamond abrasive discs with resin, bronze, and vitrified bonds were compared. The soft and tough resin-bonded disc yields the best performance, reducing surface roughness Ra from 420 nm to 106 nm. In the fine lapping stage, three metallic discs—Cu, Fe, and WC-Co—were evaluated. The high-stiffness WC-Co disc achieves the best results, with an Ra of 11.2 nm under conditions of 0.45 MPa and 600 r/min. Molecular dynamics (MD) simulations further reveal that increasing lapping pressure significantly enhances the material removal rate but concurrently aggravates subsurface damage (SSD), while the effect of speed is considerably smaller. Therefore, pressure emerges as the key parameter that requires balanced optimization in the fine lapping process.

## 1. Introduction

Diamond, as the hardest substance in nature (Mohs hardness 10), has attracted extensive research interest since its discovery. Owing to its ultra-wide bandgap, high thermal conductivity, high electrical conductivity, and exceptional chemical stability, it has been hailed by the academic community as the “material king of the 21st century” and the “ultimate semiconductor material” [1,2]. Compared with the first three generations of semiconductor materials, diamond crystals possess superior properties. These include a wider bandgap, higher breakdown electric field, higher saturated electron drift velocity, higher hole mobility, and excellent thermal stability. Therefore, diamond wafers have broad application prospects in various high-end fields such as semiconductors and optical windows [3,4].

However, the oriented growth of polycrystalline diamond (PCD) wafers is relatively difficult, as the grains are arranged in a disordered manner with sharp edges, and impurities exist at grain boundaries, which significantly reduce the surface accuracy of PCD wafers [5]. Lapping and polishing are key steps for their application. Currently, the processing methods for diamond planarization are mainly divided into contact processing and non-contact processing. Contact processing includes conventional mechanical lapping and polishing [6], chemical mechanical polishing (CMP) [7], and thermochemical polishing [8], while non-contact processing includes laser machining [9] and plasma beam polishing [10]. Thermochemical polishing is performed in a vacuum at elevated temperatures of 730–950 °C, where the friction between the metal plate (Fe, Ni) and the diamond surface during processing induces localized graphitization, which is subsequently removed by relative sliding motion. However, the excessively high processing temperature cannot guarantee the surface quality of the removed material [11]. Laser machining has high removal efficiency but poor surface accuracy, and the thermal ablation material removal method may cause thermal cracks and other damage on the diamond surface [12,13]. Plasma beam polishing can achieve nanometer-scale surface quality, but its equipment is expensive and processing costs are high, making it mainly a technical means for improving surface precision [10,11]. In contrast, mechanical lapping remains a practical approach for achieving planarization of PCD due to its simple equipment and low cost.

In recent years, scholars have conducted extensive research on the mechanical lapping process of diamond. Li et al. [14] employed loose abrasives for mechanical lapping of PCD, achieving a surface roughness Ra of approximately 96 nm on PCD slices under conditions of 0.3 MPa lapping pressure, W10 abrasive grit size, and 4% slurry concentration. Li et al. [5] utilized reactions between transition metals and diamond wafers to improve the material removal rate and particle elimination effect. The results showed that transition metals can promote the transformation of surface particles into amorphous carbon, thereby achieving superior processing results. The Fe abrasive wheel exhibited the highest material removal rate and the best ground surface morphology, achieving a surface roughness Ra of 68.4 nm. Xin [15] employed a high-speed self-rotating grinding device combined with a self-sharpening fixed abrasive diamond wheel to achieve high-precision grinding of single-crystal diamond (SCD), and investigated the material removal mechanism through morphological characterization, elemental content analysis, and other detection methods. The results indicated that the material removal mode on the diamond (100) crystal plane is influenced by cutting depth and involves a synergistic removal process along the (110) and (100) directions. Among these directions, the brittle–ductile transition along the (110) direction significantly affects the processing quality. Surface fragmentation and subsurface cracks mainly arise from cleavage along the (111) planes. Yuan et al. [16] used a diamond lapping and polishing equipment developed by Shenyang University of Technology to study the lapping and polishing performance of PCD. The results showed that when the surface roughness of PCD falls below Ra 20 nm, grain boundaries and voids become apparent. These grain boundaries and voids are the main variables causing the increase in surface roughness during mechanical polishing of chemical vapor deposition (CVD) diamond. In terms of mechanistic research, MD simulation is a commonly used atomic-scale research tool. Currently, many scholars have employed MD software to extensively study the removal and damage mechanisms during diamond processing. Xin et al. [17] used MD simulations to analyze the material removal behavior of SCD with different orientations. The results showed that the SSD on each crystal plane originates from the (111) cleavage plane, but manifests in different ways: the (100) crystal plane cleaves along the <110> direction; the (110) crystal plane cleaves simultaneously along the <110> direction and the orthogonal <112> directions; and the (111) crystal plane exhibits a combination of horizontal peeling and 60° inclined cleavage. Lin et al. [18], in order to better explain the contact and friction deformation mechanisms of diamond during nanoindentation and sliding processes, combined MD and experimental methods to study the friction and indentation behavior of diamond abrasives on diamond surfaces. The results indicated that indentation velocity is one of the main factors governing the contact indentation deformation mechanism. Lin et al. [19] employed reactive MD simulations to study the high-temperature wear mechanism of diamond, and found that there exists a critical temperature (2000–2500 °C). When this temperature is exceeded, the mechanical strength of diamond decreases significantly, and the diamond material is rapidly removed after only a sliding distance of several tens of nanometers.

The above studies provide an important foundation for understanding the lapping removal behavior of diamond, but the following shortcomings still exist. At the experimental level, most existing studies focus on SCD or only concern a specific process stage of PCD, and a systematic research framework from rough lapping to fine lapping has not yet been established. In terms of mechanistic analysis, research on the internal damage mechanisms during diamond grinding is mainly concentrated on SCD. However, due to the presence of grain boundaries in PCD, its atomic packing, crack propagation, and amorphization behavior differ fundamentally from those of SCD. Therefore, further construction of an MD model for nanoscale grinding of PCD is of great value for understanding the role of grain boundaries in the material removal process and optimizing the lapping process. In view of the above problems, this paper takes MPCVD PCD as the research object and conducts a systematic study from two aspects: abrasive tool selection and process optimization. First, three types of fixed diamond abrasive discs with typical bonds, namely resin bond (soft and tough), bronze bond (hard and strong), and vitrified bond (highly brittle), were designed and fabricated. These discs were used to systematically analyze the effects of bond hardness and brittleness on rough lapping surface integrity. Second, three metallic lapping discs—low-stiffness Cu disc, medium-stiffness Fe disc, and high-stiffness WC-Co disc—were used for fine lapping. Raman spectroscopy was used to evaluate the surface crystal quality, revealing the influence of metallic disc support stiffness on fine lapping performance. Furthermore, the influence laws of lapping speed and pressure on surface roughness were systematically investigated, and the optimal processing parameters were selected. Finally, an MD model for nanoscale grinding of PCD was constructed to elucidate, at the atomic scale, the influence mechanisms of pressure and velocity on material removal behavior and SSD. This study aims to provide systematic process guidance and theoretical basis for high-efficiency and high-quality mechanical lapping of PCD.

## 2. Experimental Preparation

### 2.1. Experimental Equipment

**Figure 1:**
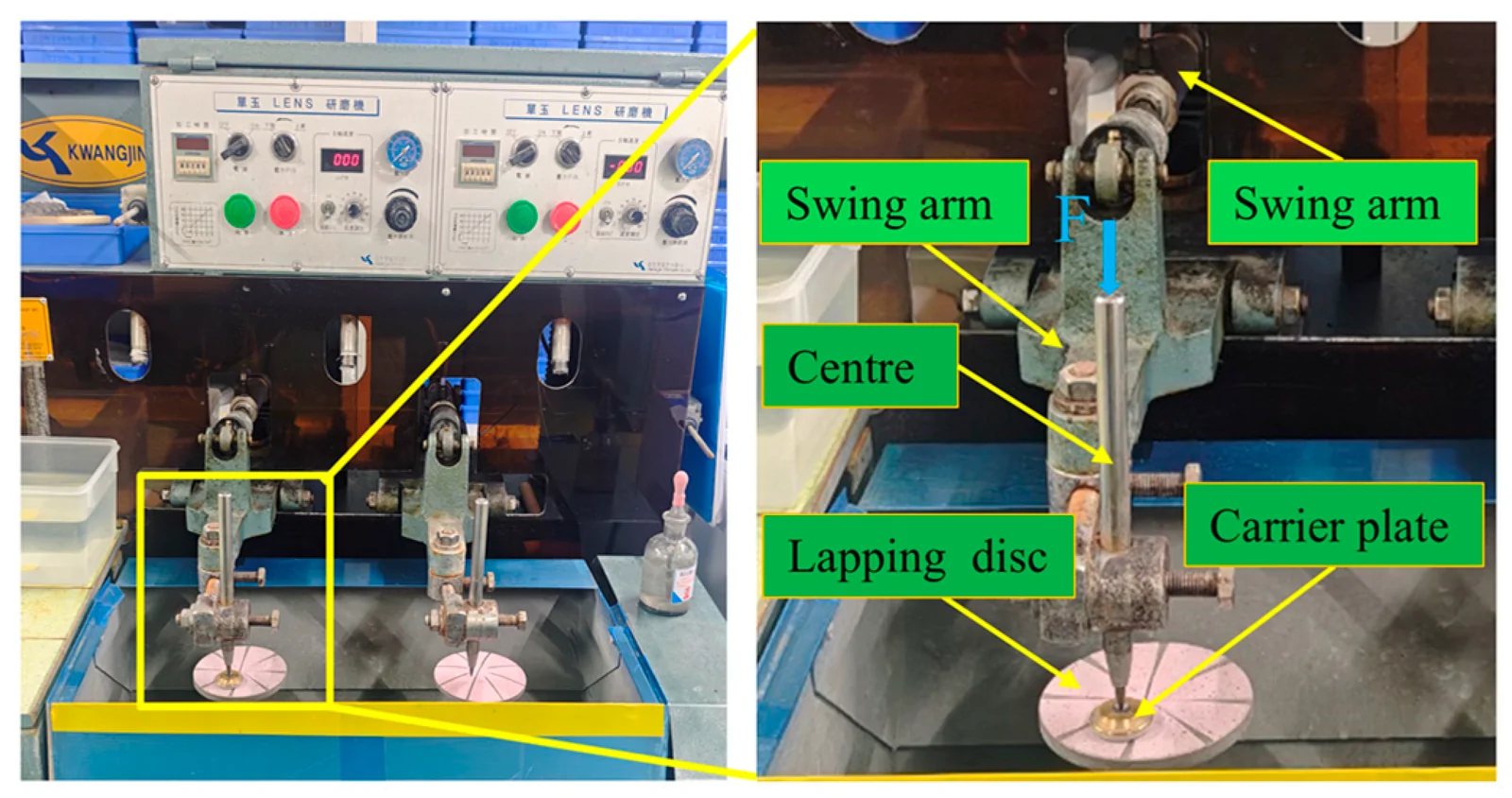
Danyu lapping machine.

The lapping experimental equipment used in this study is a Danyu lapping machine manufactured by Gwangjin Precision Co., Ltd., Changwon, Republic of Korea (as shown in Figure 1). Its spindle speed ranges from 0 to 2000 r/min, and the normal pressure is pneumatically adjustable from 0 to 0.6 MPa. Specifically, the desired pressure is directly preset on the machine’s pressure regulator, and the resulting thrust is transmitted to the specimen carrier plate through a center pin, generating the predetermined normal pressure on the lapping interface. The pressure values reported in this study refer to these preset input pressures displayed on the machine gauge. A magnetic stirrer is used to continuously agitate the slurry to maintain the dispersion of abrasive grits. Meanwhile, a peristaltic pump is employed to uniformly drip the slurry onto the lapping disc.

**Figure 2:**
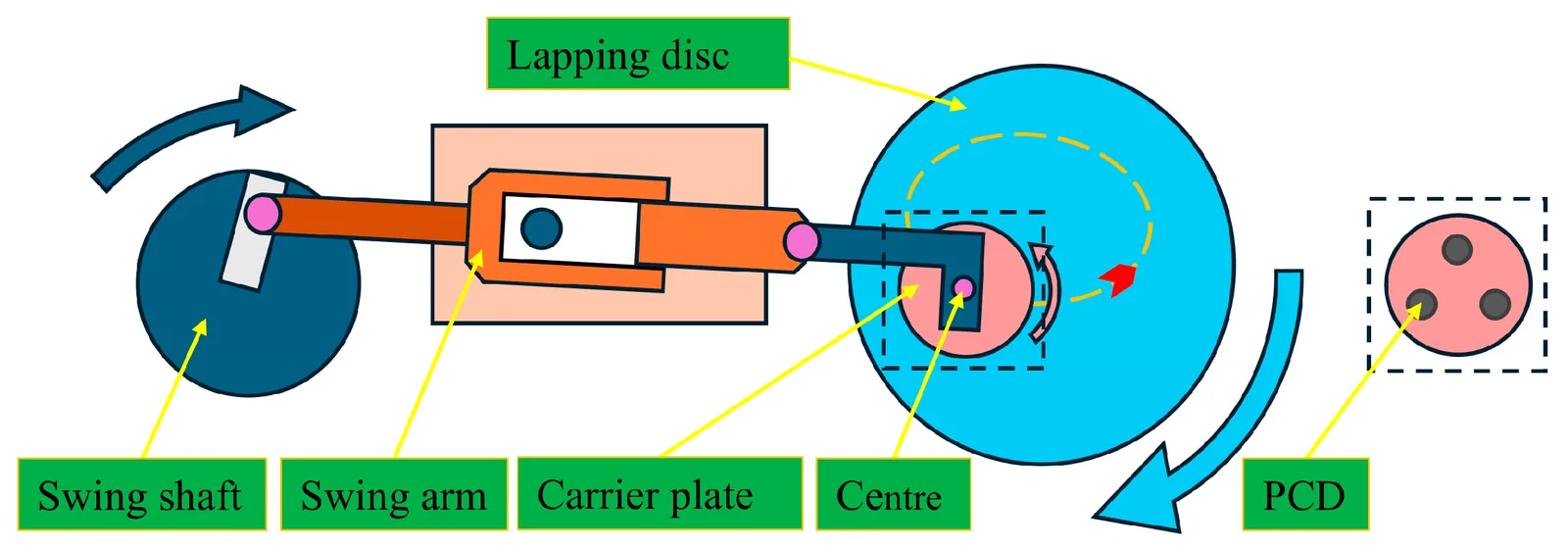
Schematic diagram of motion of the experimental equipment.

As shown in Figure 2, the lapping experimental setup achieves compound motions through multiple independent mechanisms. The spindle motor drives the lapping plate to rotate about its central axis, supplying the primary cutting velocity for material removal. The swing shaft, via the eccentric adjustment mechanism, drives the swing arm and subsequently the carrier plate to perform reciprocating oscillation along the radial direction of the lapping plate, ensuring uniform crossover coverage of the lapping trajectories. Meanwhile, the carrier plate is driven by frictional contact with the lapping plate, allowing free rotation during processing, which further diversifies the trajectory distribution. These motions are realized through independent power sources or driving modes and are coordinately controlled by the control system to collectively accomplish the complex trajectory-based lapping process.

**Figure 3:**
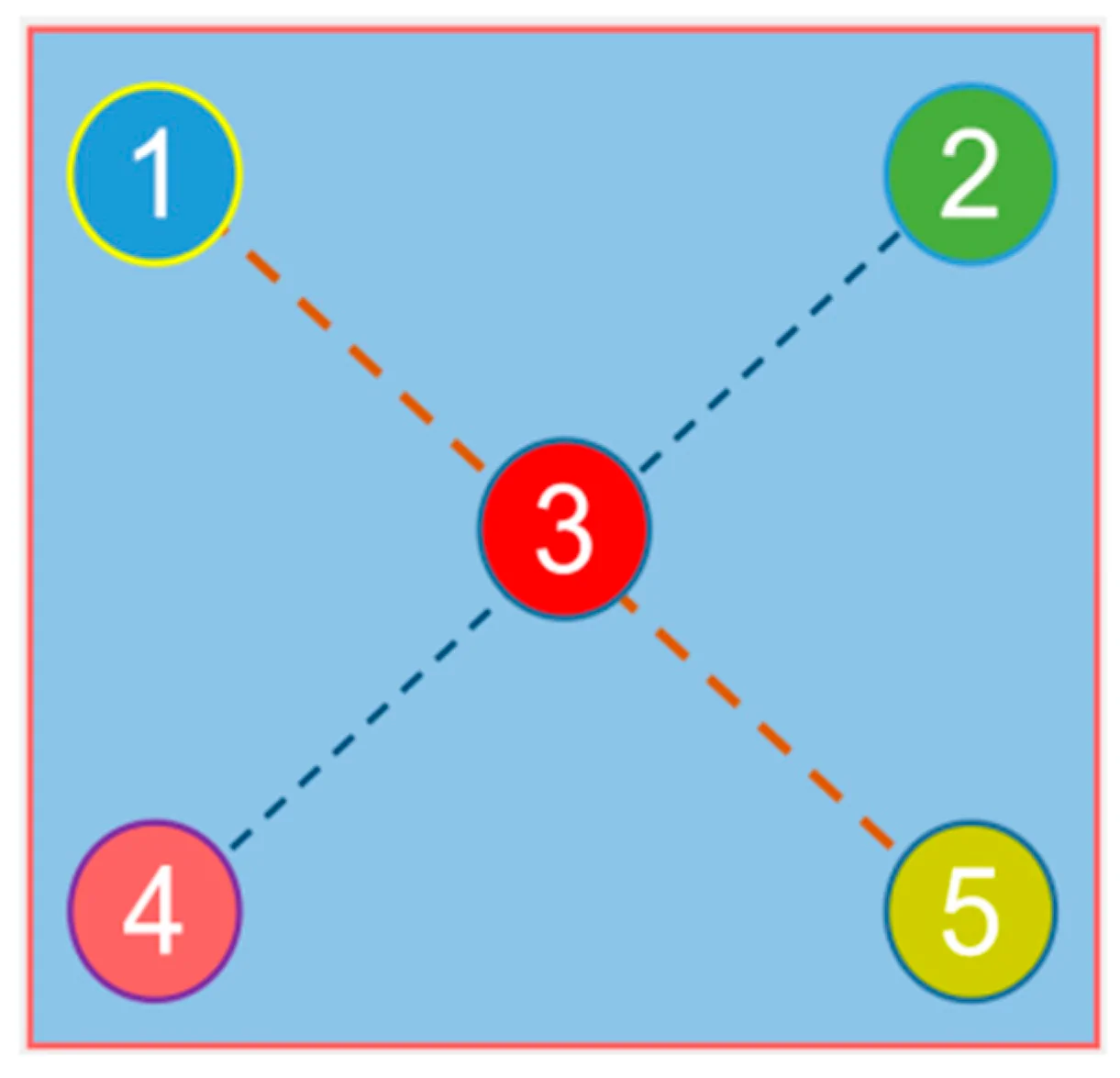
Schematic of measurement point picking.

To facilitate direct comparison with previous studies, Ra values—a widely adopted and standardized parameter in the precision lapping field—were measured using a Newview 8300 white light interferometer (WLI, Zygo Corporation, Middlefield, CT, USA), and the surface morphology was also observed. The measurement range was 416 μm × 416 μm. Five points were measured on the specimen surface and the average value was taken, with the point selection method shown in Figure 3. A BX metallurgical microscope manufactured by Olympus Corporation, Tokyo, Japan, was employed to observe the microscopic surface of the PCD, with a 200× objective lens under differential interference contrast (DIC) mode. A Horiba LabRAM Odyssey Raman spectrometer (HORIBA France SAS, Palaiseau, France) with a laser wavelength of 532 nm was used to analyze the compositional changes on the PCD surface.

### 2.2. Experimental Specimens

**Figure 4:**
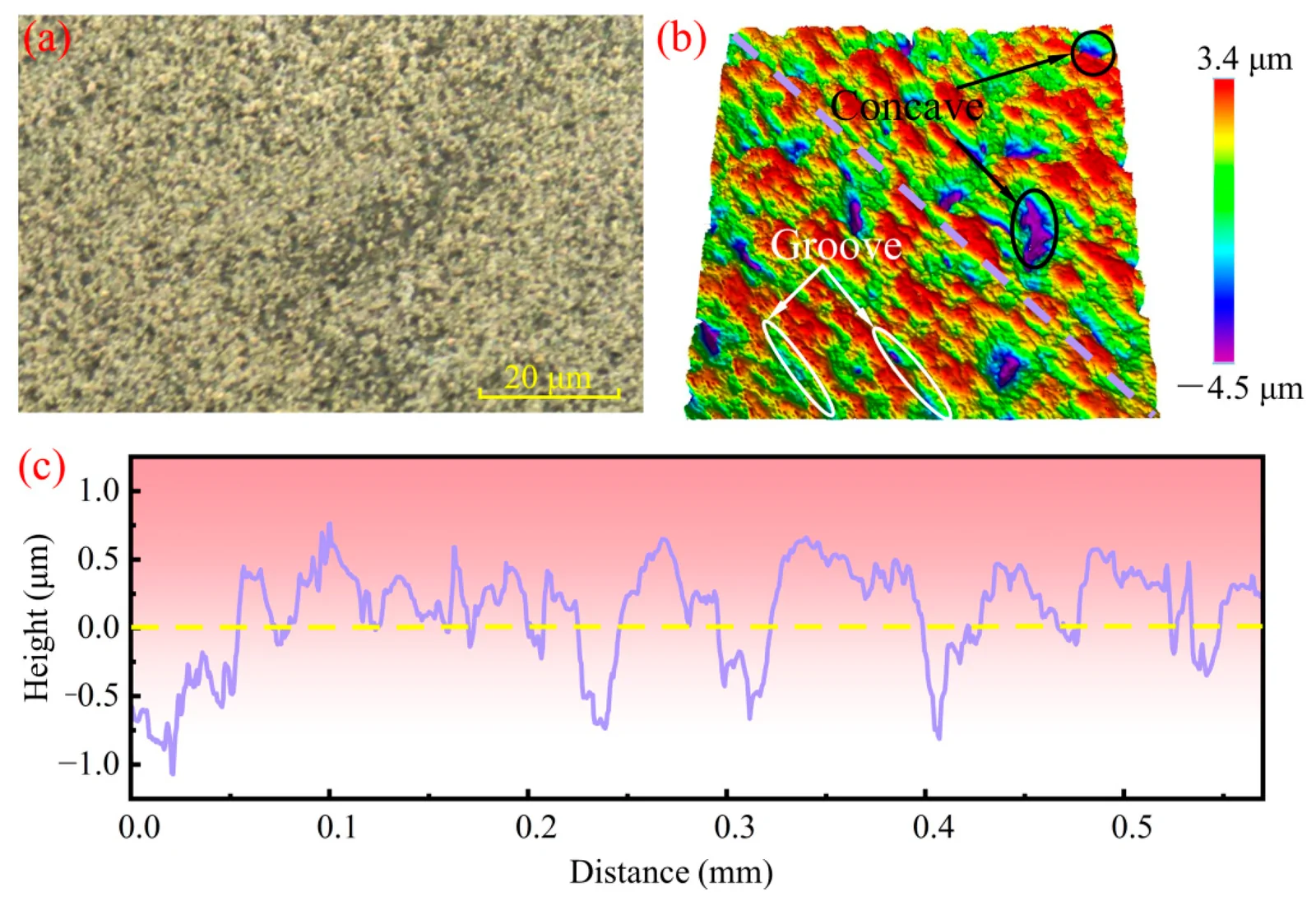
Optical micrograph (**a**), 3D surface topography (**b**), and diagonal surface profile (**c**) of the PCD specimen.

To reduce experimental costs, the specimens used in this study were PCD with a radius of 2.5 mm. Their surfaces were pretreated by double-sided milling and grinding, with an initial surface roughness Ra of approximately 420 nm. Figure 4a shows the initial surface morphology observed under an Olympus metallurgical microscope, revealing a poor surface finish. Figure 4b and Figure 4c present the three-dimensional surface topography obtained by WLI and the corresponding diagonal cross-sectional profile, respectively. The three-dimensional surface morphology exhibits poor flatness with numerous defects such as pits and grooves. The diagonal profile shows significant fluctuations, with five valleys deeper than −0.5 μm (one of which exceeds −1 μm) and five peaks higher than 0.5 μm. The peaks and valleys are pronounced and relatively broad in width.

## 3. Design, Fabrication, and Rough Lapping Performance Analysis of Diamond Lapping Discs

### 3.1. Fabrication of Diamond Lapping Discs

**Figure 5:**
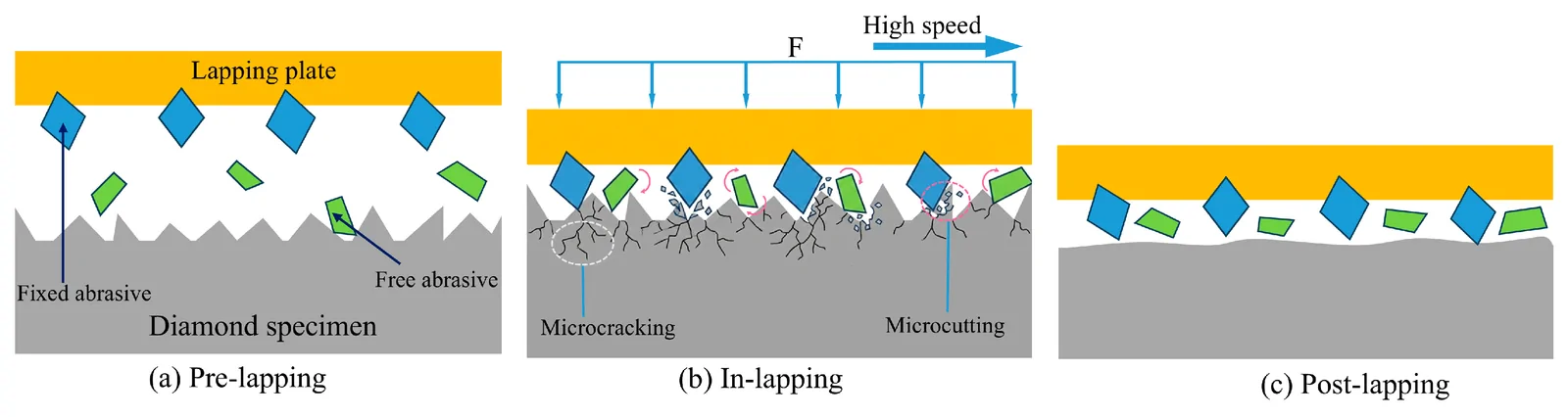
Schematic diagram of the material removal mechanism.

Mechanical lapping primarily removes material by inducing microfracture on the diamond surface through mechanical action. At high rotational speeds, if loose abrasives are directly used for polishing, the abrasive grits are rapidly thrown off and cannot effectively perform cutting on the workpiece surface, significantly reducing the processing efficiency. Therefore, under high-speed lapping conditions, this study adopts a dual-action approach combining fixed abrasives and loose abrasives to achieve efficient material removal of diamond. The schematic diagram of the material removal mechanism is shown in Figure 5. At high rotational speeds, loose abrasives cause microfracture pits on the diamond surface through impact and rolling actions, thereby achieving material removal. Meanwhile, fixed abrasives mainly cut into the workpiece surface at a fixed negative rake angle, forming high-speed micro-cutting and thereby achieving micro-removal of the diamond surface material.

**Figure 6:**
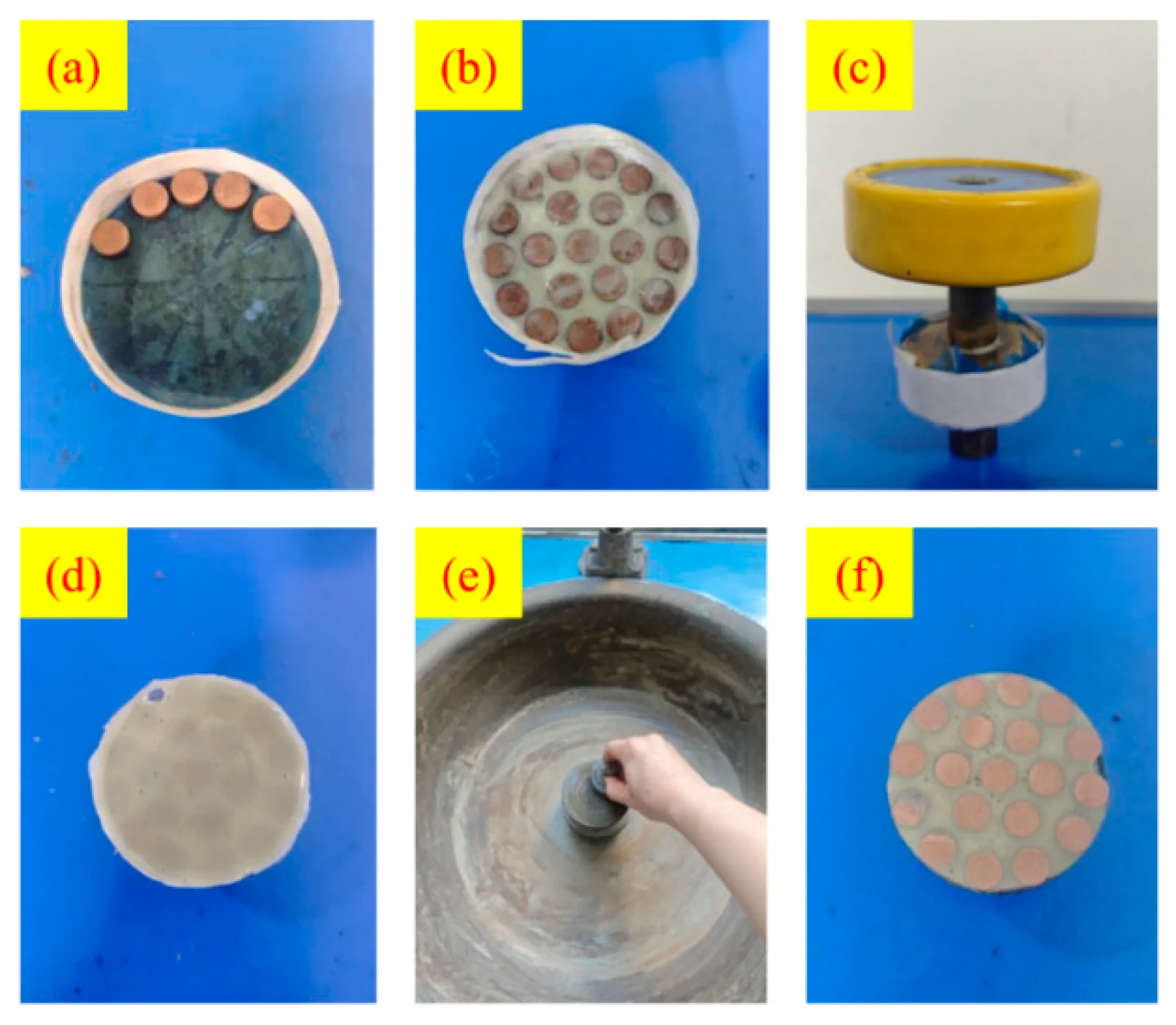
Manufacturing process of the diamond lapping plate: (**a**) pellet arrangement; (**b**) adhesive filling; (**c**) pressing; (**d**) curing; (**e**) planarizing; (**f**) finished product.

However, traditional fixed diamond lapping pads have high costs and significant wear, making them unsuitable for high-speed lapping of PCD. Therefore, three types of diamond pellets with stronger wear resistance—resin bond, bronze bond, and vitrified bond—were selected to design and fabricate three different fixed diamond lapping discs in which the diamond abrasive grit size was W10. The specific fabrication process is shown in Figure 6. First, the diamond pellets were arranged in a certain order and bonded onto a flat lapping plate. Then, epoxy resin adhesive was slowly applied to fully fill the edge gaps between the pellets. The filling process was carried out as uniformly as possible to avoid bubble generation, as shown in Figure 6a,b. After the adhesive filling was completed, an iron weight was placed on top to press and flatten the assembly, which was then left to stand at room temperature for 12 h to allow sufficient curing, as shown in Figure 6c,d. Subsequently, a surface planarizing machine was used to level the rough surface of the lapping disc, during which the flatness of the disc was ensured, as shown in Figure 6e. Finally, the disc was repeatedly rinsed with deionized water and then blown dry with a high-pressure air gun to prevent the incorporation of coarse abrasive powder, which could otherwise affect the surface quality of the processed specimens.

### 3.2. Screening Analysis of the Effects of Bond Hardness and Brittleness on Lapping Performance

**Figure 7:**
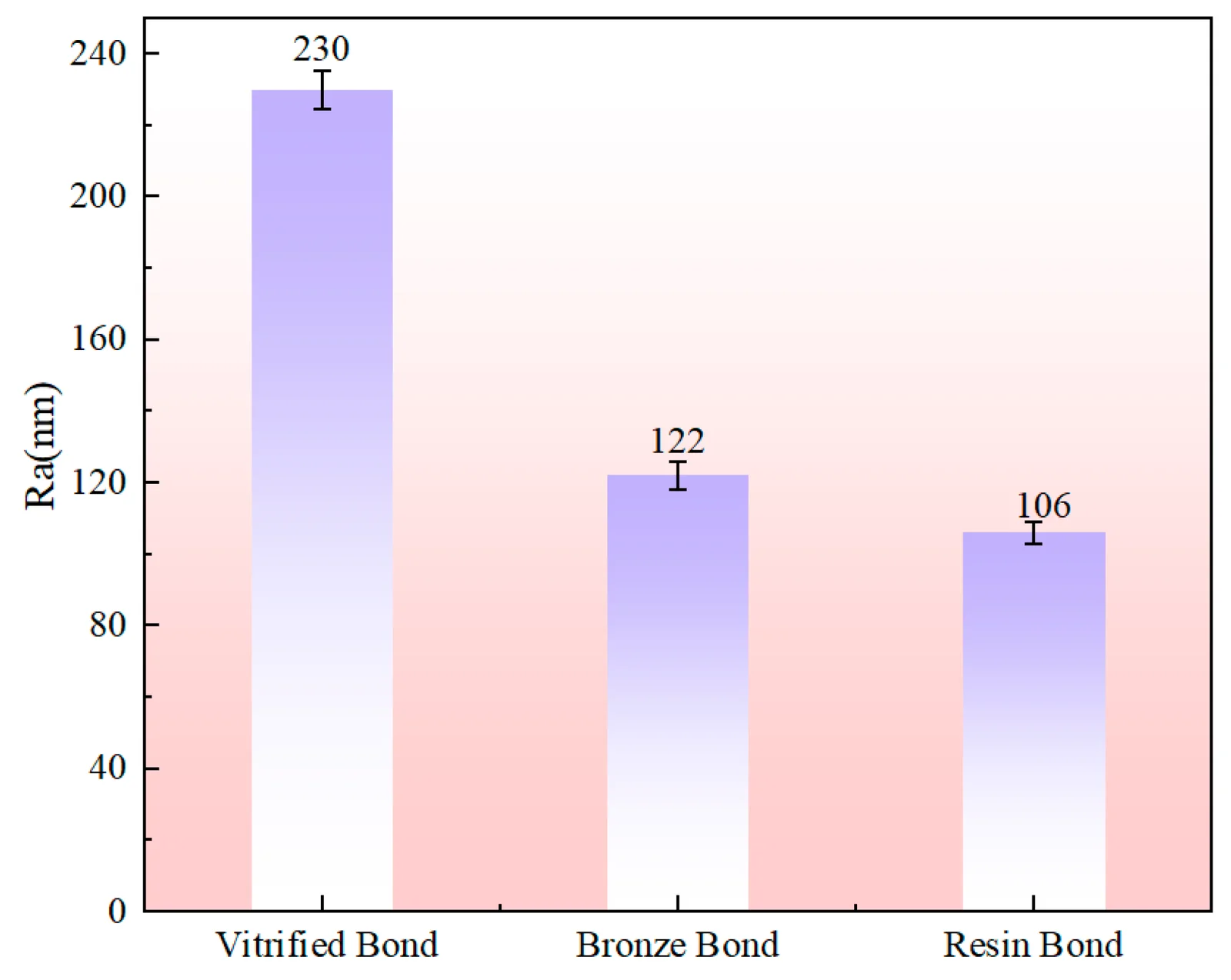
Surface roughness of workpieces rough-ground with different bond types.

To investigate the effects of bond hardness and brittleness on the rough lapping surface integrity of PCD, three typical bonded diamond lapping discs—resin bond, bronze bond, and vitrified bond—were selected for comparative experiments. The aim was to identify the most suitable bond type for rough lapping of this hard and brittle material. To avoid wafer chipping and edge breakage caused by excessive lapping pressure, relatively safe pressure and rotational speed parameters were adopted: a pressure of 0.45 MPa, a rotational speed of 1200 r/min, W10 SCD abrasive grits, a grit concentration of 45 g/L, and a lapping duration of 16 h. The surface roughness of the specimens after rough lapping with different bonds is shown in Figure 7. After lapping with the bronze-bonded disc, the surface roughness decreased from the initial 420 nm to 122 nm, a reduction of 70.95%. With the resin-bonded disc, it decreased to 106 nm, a reduction of 74.76%. With the vitrified-bonded disc, it only decreased to 230 nm, a reduction of 45.24%. Among the three bond types, the soft and tough resin-bonded disc exhibited the best rough lapping performance, followed by the hard and strong bronze-bonded disc with high holding force, while the highly brittle vitrified-bonded disc performed the worst.

**Figure 8:**
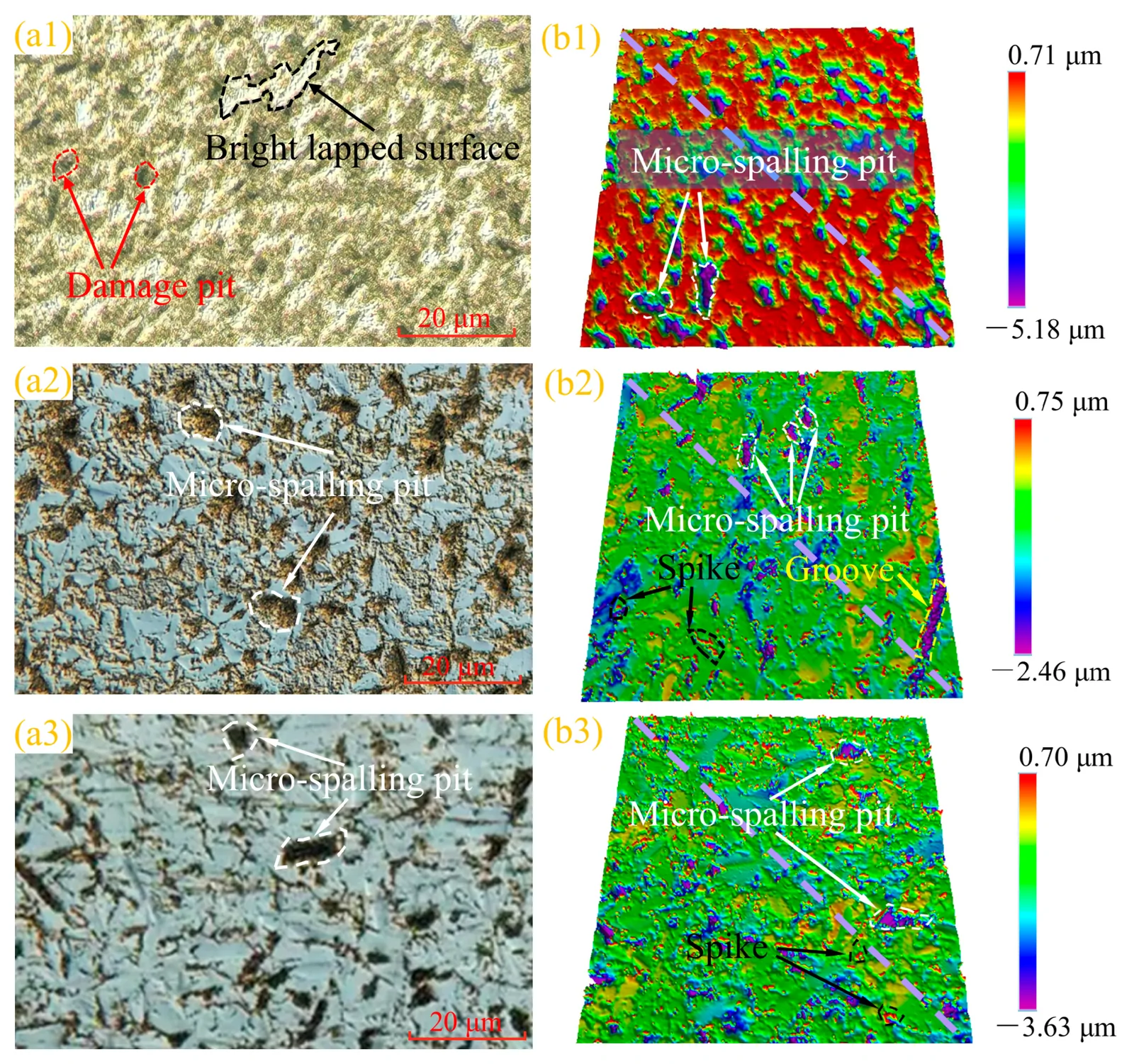
Optical surface morphologies and three-dimensional surface morphologies of surfaces machined by vitrified bond (**a1**,**b1**), bronze bond (**a2**,**b2**), and resin bond (**a3**,**b3**) lapping wheels.

**Figure 9:**
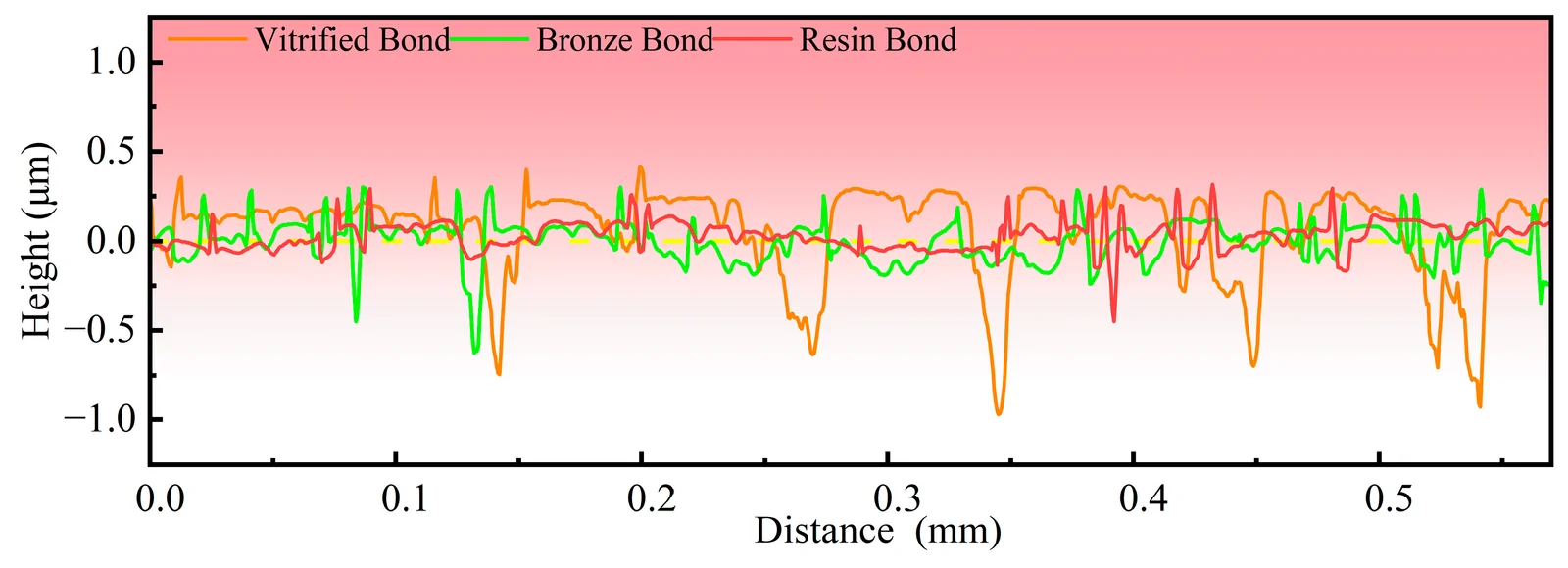
Diagonal cross-sectional profiles of workpieces ground with different bonded abrasive discs, corresponding to the dashed line positions in Figure 8.

Figure 8 and Figure 9 show the surface micromorphology and the diagonal cross-sectional profiles corresponding to the three-dimensional surface topography of the specimens after rough lapping with different bonded discs, respectively. For the vitrified-bonded disc, the optical surface morphology shows obvious polished regions, but the overall flatness is poor with numerous pit defects. The three-dimensional topography reveals a large number of pits scattered across the surface, exhibiting the worst surface flatness. The diagonal profile shows significant fluctuations with multiple valleys deeper than −0.5 μm and relatively broad peak widths. This may be attributed to the high brittleness and large elastic modulus of the vitrified bond. When abrasive grits are excessively loaded, the bond cannot buffer the impact load through its own micro-deformation. As a result, the abrasive grits cut into the workpiece at a large negative rake angle, inducing brittle fracture on the diamond surface and forming wide and deep micro-spalling pits. After lapping with the bronze-bonded disc, the optical surface morphology shows significantly improved surface quality, but numerous pits caused by mechanical microfracture still remain. The three-dimensional topography reveals many pits, grooves, and unremoved sharp peaks. Although no valley deeper than −1 μm is observed in the diagonal profile, densely distributed sharp peaks with a width of approximately 0.25 μm are present overall, indicating that the excessively hard bronze bond causes forced ploughing by blunt abrasive grits, generating a large number of micro-peaks. In contrast, although the resin-bonded disc still leaves some microfracture pits in the micromorphology, the sharp peak phenomenon is significantly improved in the three-dimensional topography. The diagonal profile shows smooth fluctuations in the first half with only sporadic narrow peaks, and although valleys exist in the second half, they are relatively shallow, with sparse peaks overall. This is mainly because the resin bond has low hardness and good elasticity, resulting in weak holding force on abrasive grits. Blunt grits are promptly dislodged while fresh sharp grits are continuously exposed, allowing the material removal to tend toward plastic or semi-plastic behavior, thereby obtaining the smoothest surface with the fewest sharp peaks. Based on the comprehensive evaluation of roughness and micromorphology, the highly brittle vitrified bond and the excessively hard bronze bond are excluded, and the soft and tough resin-bonded diamond lapping disc is selected as the optimal solution for rough lapping of PCD.

## 4. Screening Analysis of Metallic Disc Support Stiffness on Fine Lapping Performance

**Figure 10:**
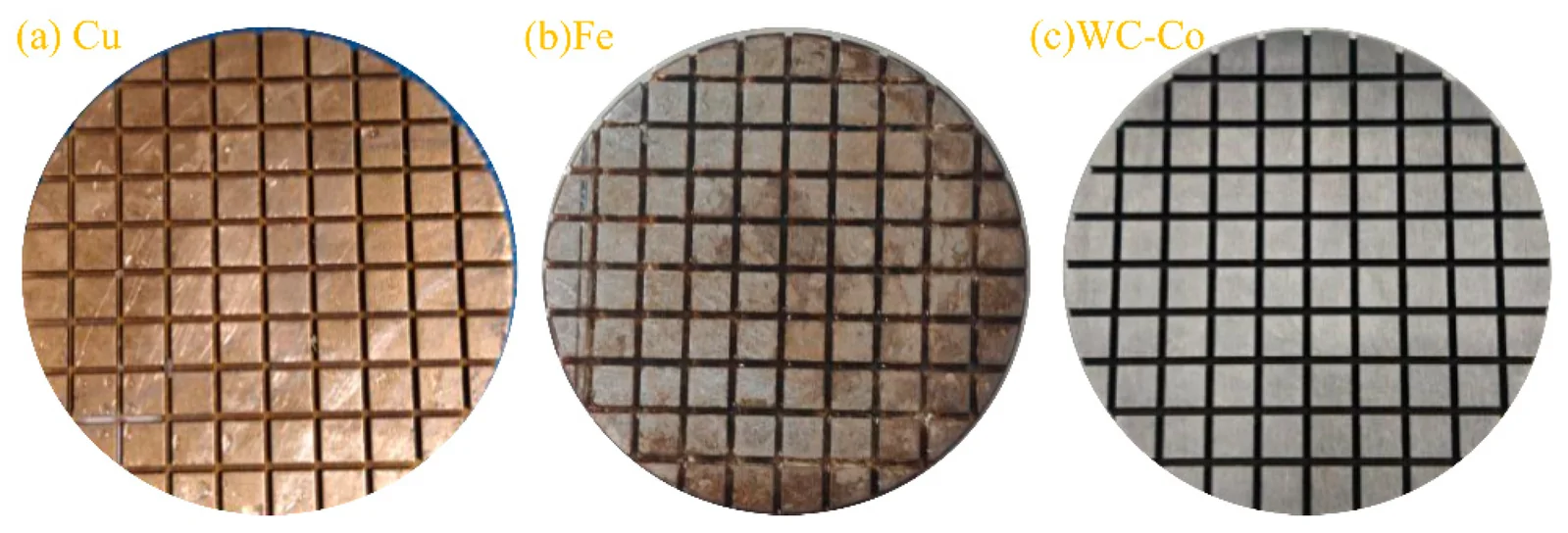
Three types of metal lapping discs.

After rough lapping of PCD with the W10 diamond lapping disc in the preceding section, a large number of pits and sharp peaks caused by mechanical microfracture still remained on the surface, with a roughness Ra of approximately 106 nm. Therefore, further fine lapping treatment was required to effectively remove the defective layer and reduce the surface roughness. In the fine lapping stage, the self-sharpening property of the resin bond relied upon during rough lapping was no longer needed. Instead, the lapping disc was required to provide stable and uniform support for fine abrasive grits, thereby achieving fixed-depth micro-cutting. To this end, three metallic lapping discs of different materials were designed, and square grid grooves were carved on the disc surfaces to enhance the cutting action (Figure 10). The three materials were a Cu disc (softest, HB ~ 40–60, elastic modulus ~ 110 GPa), an Fe disc (medium hardness, HB ~ 180–220, elastic modulus ~ 150–170 GPa), and a WC-Co disc (hardest, HRC ~ 88–92, elastic modulus ~ 500–600 GPa). During the fine lapping process, W3.5 SCD abrasive grits were used at a concentration of 45 g/L, with a lapping speed of 500 r/min, a lapping pressure of 0.35 MPa, and a lapping duration of 12 h.

**Figure 11:**
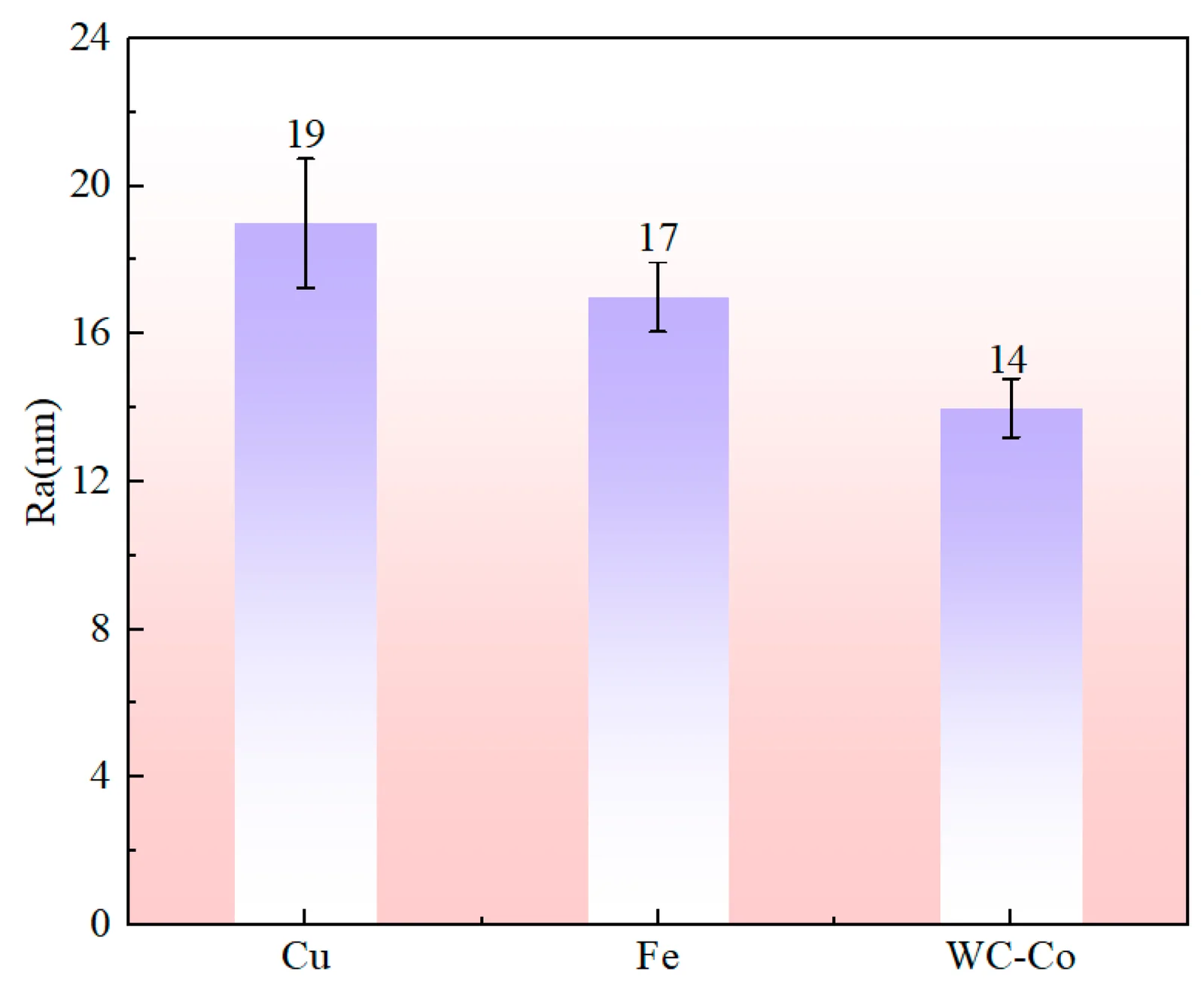
Surface roughness of specimens after lapping with different metal lapping discs.

The surface roughness of specimens after lapping with different metallic discs is shown in Figure 11. The Cu disc reduced Ra from 106 nm to 19 nm (a reduction of 82.08%), the Fe disc reduced it to 17 nm (a reduction of 83.96%), and the WC–Co disc reduced it to 14 nm (a reduction of 86.79%). Among the three metallic discs, the WC–Co disc exhibited the lowest roughness, followed by the Fe disc, while the Cu disc showed the highest roughness. This is mainly attributable to the following reasons: the soft Cu disc has insufficient stiffness and is prone to plastic deformation under pressure, causing abrasive grits to become embedded in the disc surface or roll. This behavior leads to uneven cutting depths—over-cutting in some areas and under-cutting in others—thus leaving a higher residual roughness. In contrast, the hard WC–Co disc possesses good stiffness and minimal elastic deformation, enabling abrasive grits to be firmly pressed against the workpiece surface to achieve uniform micro-cutting, thereby obtaining the lowest roughness. However, it should be noted that the absolute differences in roughness among the three discs are relatively small (within 5 nm). Therefore, roughness data alone are insufficient for a comprehensive evaluation of the overall lapping performance of the three discs, and further assessment combining surface morphology and Raman spectroscopy is required.

**Figure 12:**
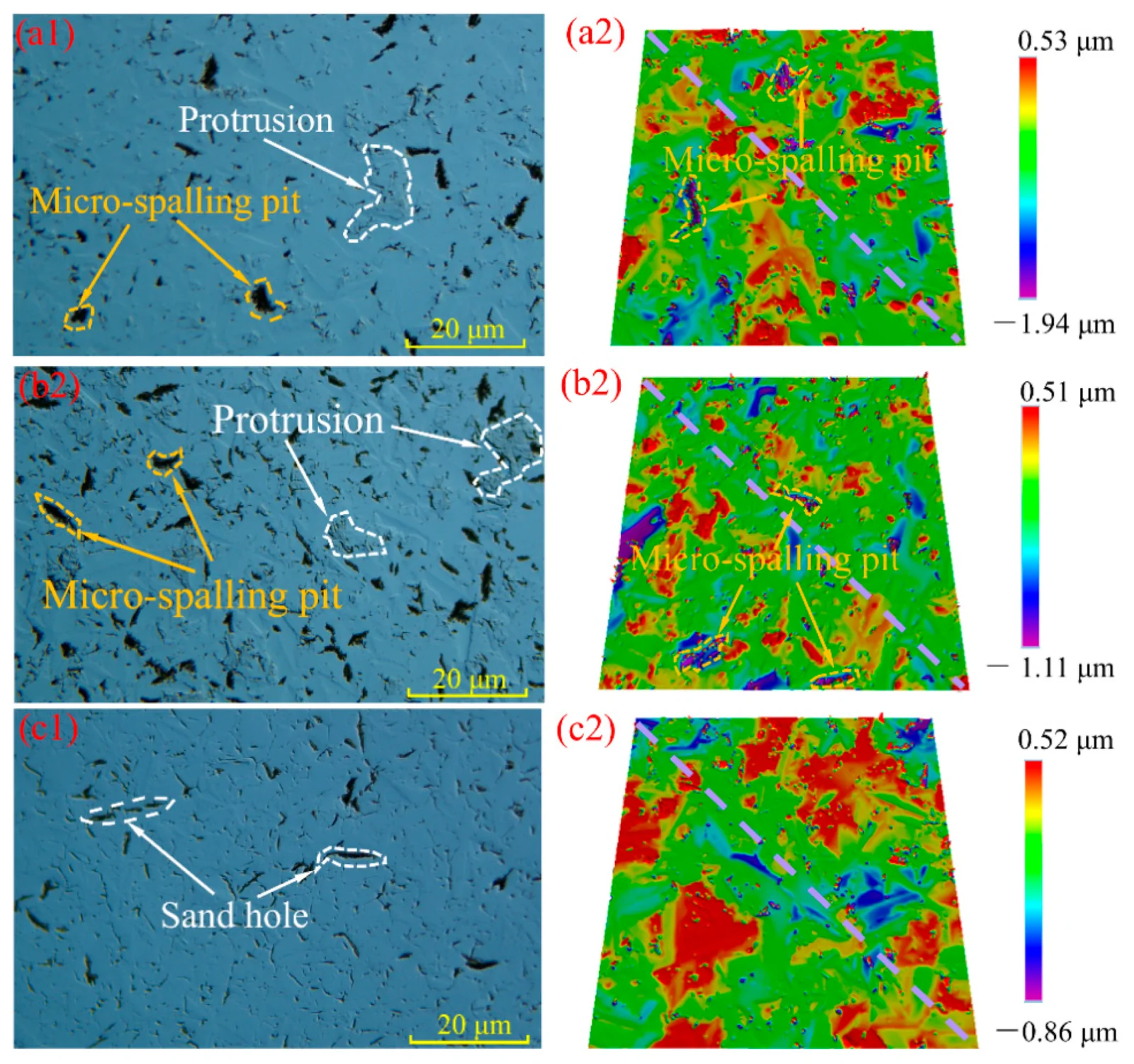
Shows the optical surface morphology and three-dimensional surface morphology of the specimen surfaces after processing with Cu disc (**a1**,**a2**), Fe disc (**b1**,**b2**), and WC-Co disc (**c1**,**c2**).

**Figure 13:**
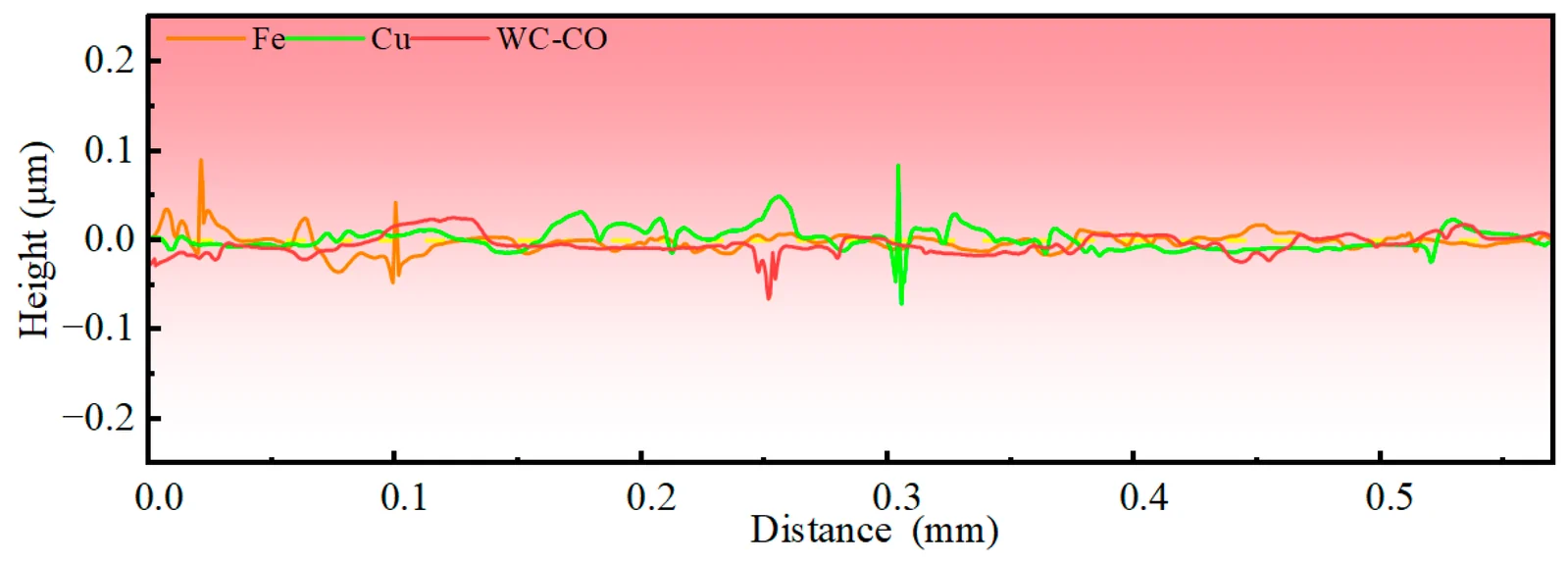
Diagonal cross-sectional profiles of workpieces ground with different metal lapping discs, corresponding to the dashed line positions in Figure 12.

Figure 12 and Figure 13 show the surface micromorphology and the diagonal cross-sectional profiles corresponding to the three-dimensional surface topography of the specimens after lapping with different metallic discs, respectively. After fine lapping with the Cu disc, numerous pits and protrusions still remained in both the optical micrographs and the three-dimensional topography. The diagonal profile exhibits significant fluctuations in the middle section, with sharp narrow peaks higher than 0.06 μm and narrow valleys deeper than −0.06 μm, indicating that the soft disc could not effectively remove the deep pits left by rough lapping, and its own non-uniform cutting introduced new sharp peaks. The Fe disc, with medium hardness, exhibited a number of micro-peaks on the surface similar to that of the Cu disc. The profile still shows peaks higher than 0.06 μm and valleys deeper than −0.04 μm in the first half, but the fluctuations in the second half are slightly smoother, indicating that the medium hardness slightly improved the defect removal capability, though not thoroughly. In contrast, the WC-Co disc shows a significant reduction in the number of pits in both optical micrographs and three-dimensional topography, with no obvious micro-protrusions. The diagonal profile exhibits the smallest fluctuation range, with only one sharp narrow valley deeper than −0.06 μm in the middle and no obvious peaks. This indicates that the high-hardness WC-Co disc, by virtue of its excellent stiffness, enables the W3.5 diamond abrasive grits to stably scrape at a constant cutting depth, effectively smoothing the sharp peaks left by rough lapping while gradually leveling the bottoms of pits, leaving only a small number of tiny pinhole-like defects. Therefore, the hardness of the metallic disc directly determines the removal efficiency of defects such as pits and sharp peaks, as well as the final surface integrity, by altering the support stiffness of abrasive grits and the uniformity of the cutting trajectory.

**Figure 14:**
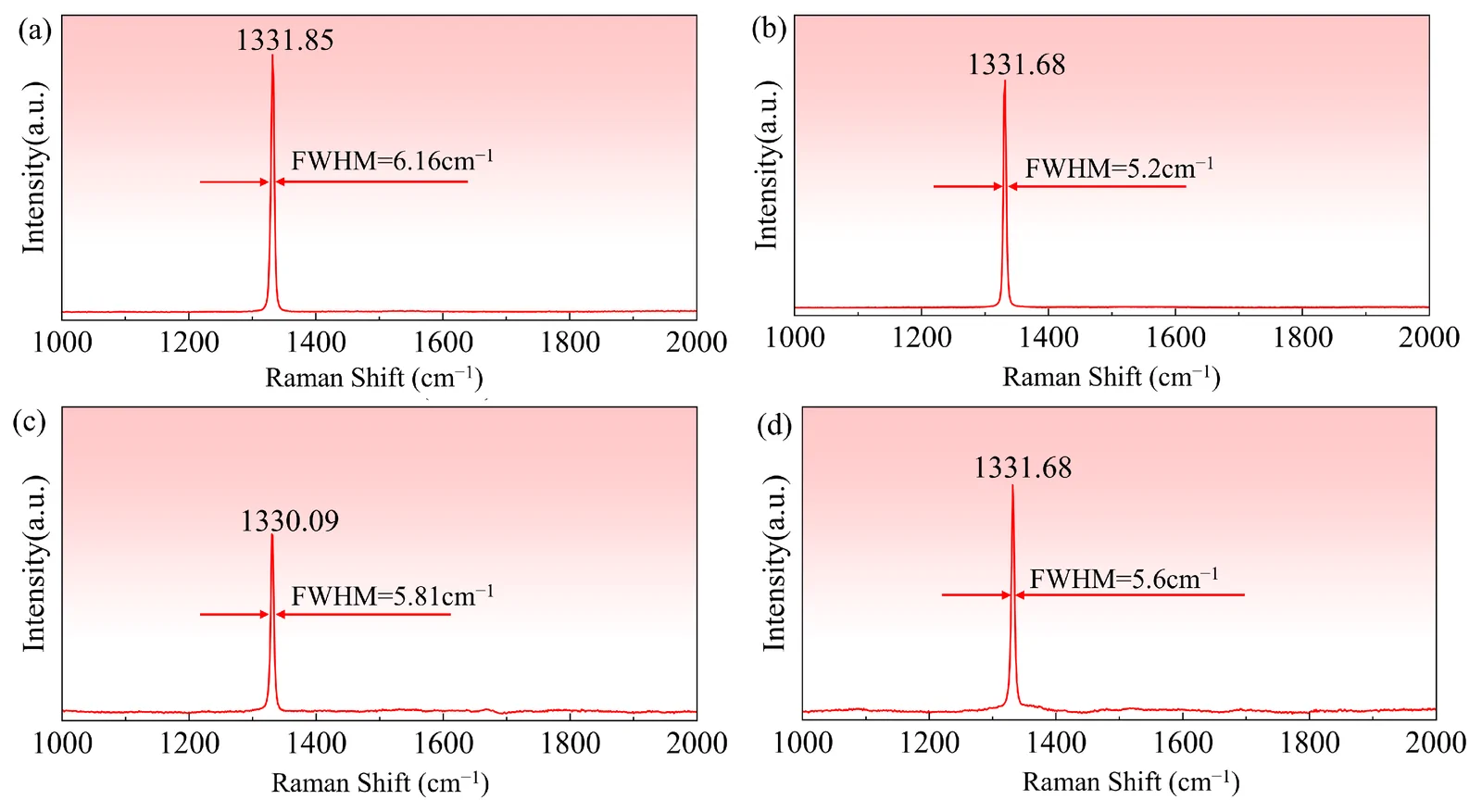
Raman spectra of PCD after rough lapping and lapping with different metal plates: (**a**) rough ground; (**b**) WC-Co disc; (**c**) Cu disc; (**d**) Fe disc.

To quantitatively evaluate the improvement effect of different lapping discs on the surface crystal integrity of diamond, Raman spectroscopy was employed for characterization in this study. Raman spectroscopy is highly sensitive to the hybridized bond types (sp^3^/sp^2^) of carbon materials, and can effectively distinguish the intrinsic diamond phase from the amorphous carbon damaged layer. Moreover, the full width at half maximum (FWHM) of the characteristic peak can semi-quantitatively reflect the surface lattice ordering. Figure 14 shows the Raman spectra of the specimens after lapping with different metallic discs. The FWHM values after fine lapping with different metallic discs, calculated by Gaussian–Lorentzian fitting, were all reduced compared with those after rough lapping. This is because the amorphous phase on the diamond surface was effectively removed after fine lapping, resulting in a more regular surface crystal structure. Among them, the WC-Co disc yielded the smallest FWHM value of 5.2 cm^−1^, which is 0.61 cm^−1^ and 0.4 cm^−1^ lower than those of the Cu disc and the Fe disc, respectively. Therefore, the WC-Co disc can achieve better surface crystal quality and was selected as the fine lapping disc for PCD.

## 5. Effect of Fine Lapping Process Parameters

Based on the results of roughness, surface morphology, and Raman spectroscopic analysis in the preceding sections, the WC-Co disc was selected as the fine lapping tool for PCD. However, the disc material is only one of the fundamental conditions for achieving high-quality fine lapping. In actual processing, lapping speed and lapping pressure also significantly affect the cutting behavior of abrasive grits and the material removal effect: if the speed is too low, the cutting frequency is insufficient, resulting in low efficiency; if the speed is too high, the abrasive grits are easily thrown off by centrifugal force, weakening the cutting action; if the pressure is too low, the micro-cutting depth is insufficient, making it difficult to remove the defective layer; if the pressure is too high, SSD may be aggravated or even wafer breakage may occur. Therefore, on the basis of the selected WC-Co disc, it is necessary to further systematically optimize the above two key process parameters to determine the optimal process parameters for the fine lapping process.

### 5.1. Effect of Different Lapping Speeds on Fine Lapping Performance

**Figure 15:**
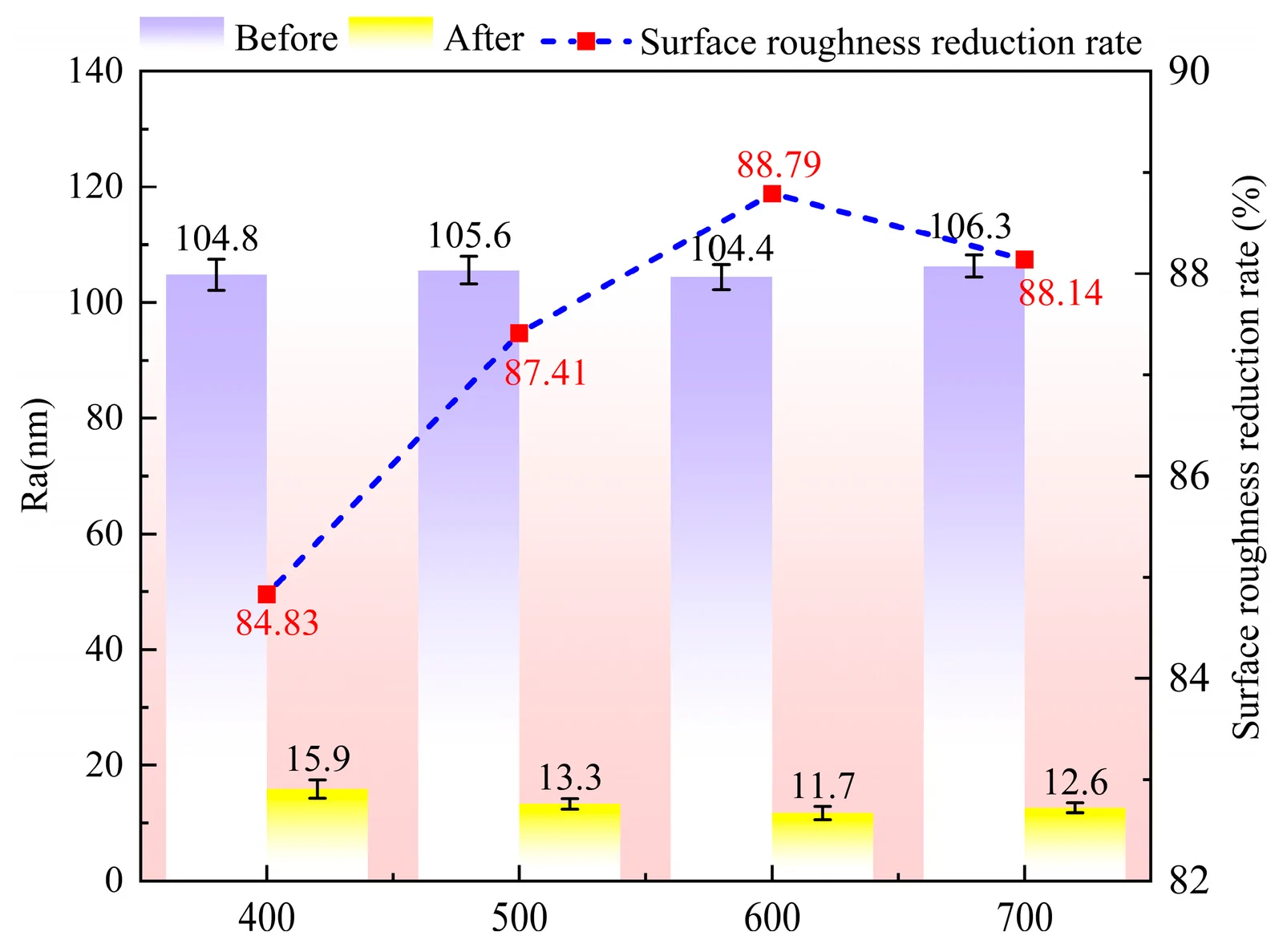
Shows the optical micrographs at different lapping speeds.

To investigate the effect of different lapping speeds on the fine lapping performance of PCD, four groups of lapping speeds—400 r/min, 500 r/min, 600 r/min, and 700 r/min—were selected for fine lapping experiments on PCD with a duration of 12 h, while the pressure was kept constant at 0.4 MPa and the abrasive grit size was W3.5. Figure 15 shows the surface roughness of the specimens before and after processing under different lapping speeds. As the lapping speed increased from 400 r/min to 700 r/min, the corresponding surface roughness Ra values of the diamond specimens were 15.9 nm, 13.3 nm, 11.7 nm, and 12.6 nm, respectively, indicating that the surface roughness first decreased and then increased with increasing lapping speed. When the lapping speed increased from 400 r/min to 600 r/min, the number of contacts and cutting events between the abrasive grits and the specimen increased continuously with the rising speed. The increase enabled more effective removal of pits and protrusions left from rough lapping. The surface roughness improvement rates of the specimens were 84.83%, 87.41%, and 88.79%, respectively. When the lapping speed reached 700 r/min, the surface roughness improvement rate decreased. This may be attributed to the excessively high speed causing a large number of loose abrasives to be thrown off, reducing the impact frequency on the specimen and thus preventing effective removal of surface defects.

**Figure 16:**
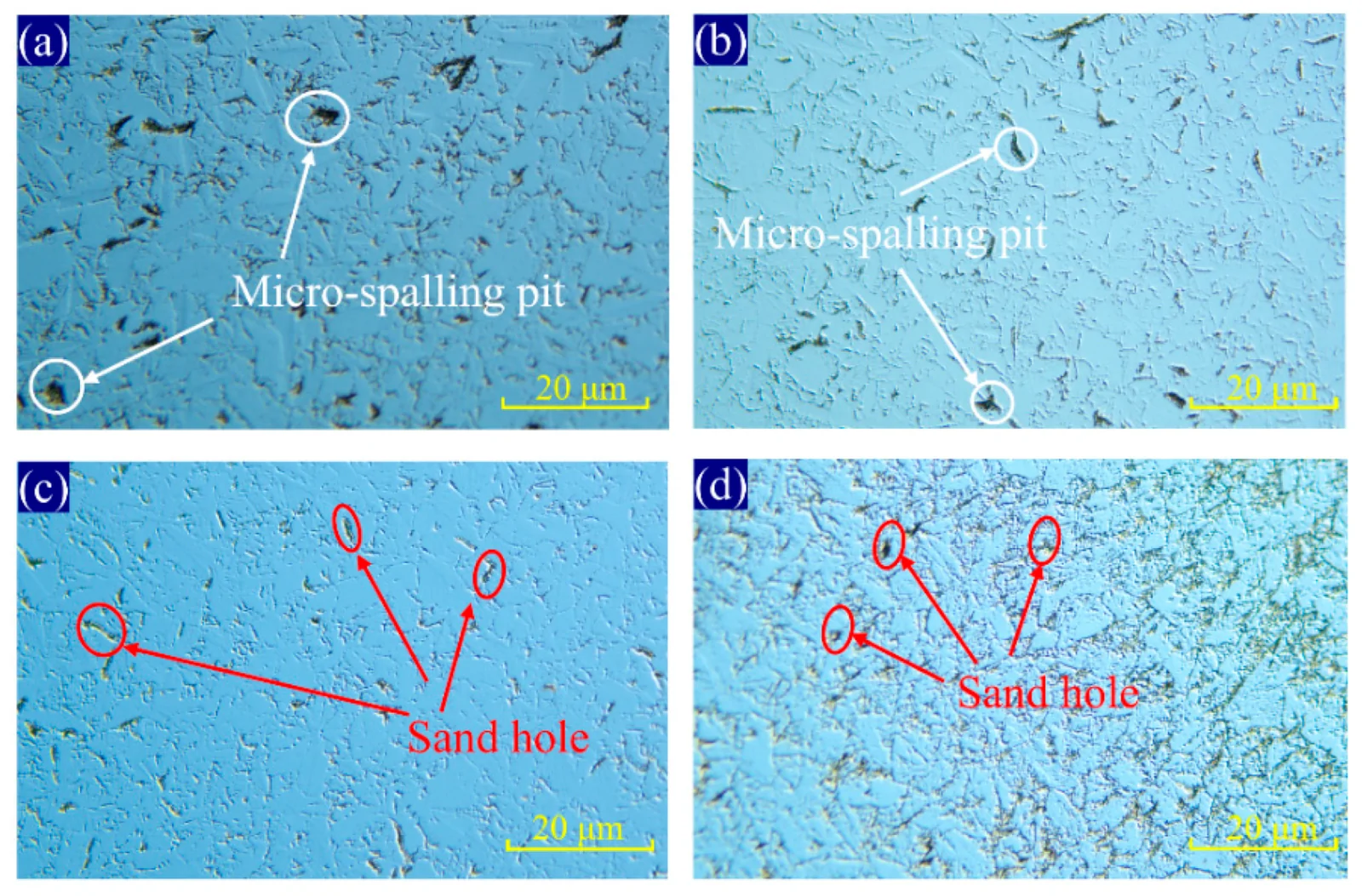
Optical micrographs under various lapping speeds: (**a**) 400 r/min; (**b**) 500 r/min; (**c**) 600 r/min; (**d**) 700 r/min.

Figure 16 shows the optical micrographs under different lapping speeds. When the lapping speed increased from 400 r/min to 600 r/min, surface defects decreased with increasing speed. When the speed was below 600 r/min, surface defects mainly consisted of relatively large micro-spalling pits. When the speed reached 600 r/min, surface defects were predominantly small sand holes. When the speed exceeded 600 r/min, the abrasive grits were easily thrown off the lapping disc under the action of centrifugal force, resulting in insufficient lapping action on the diamond surface, deteriorated surface quality, and a rough surface.

**Figure 17:**
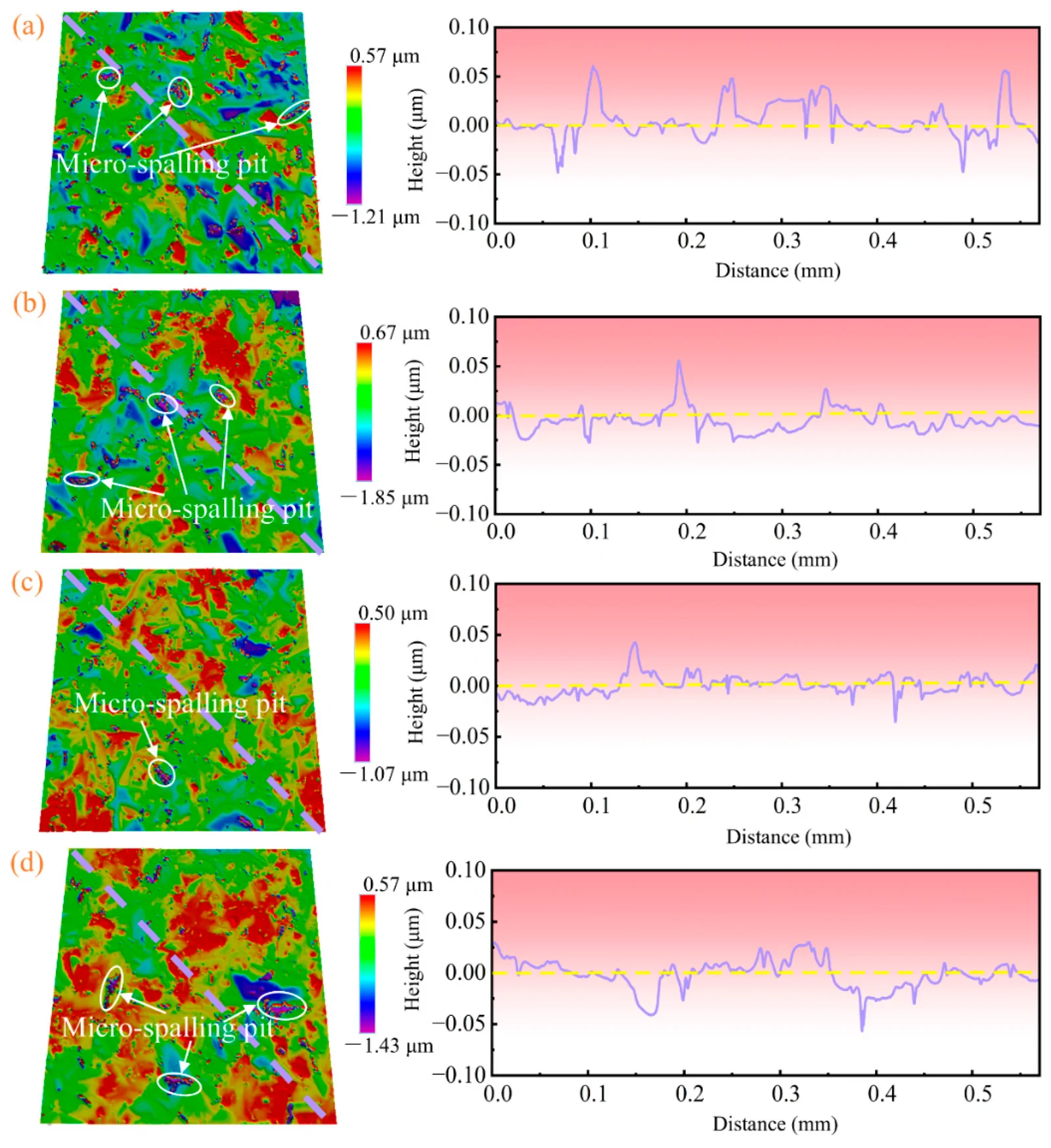
3D surface topography and diagonal profiles of diamond specimens under various lapping speeds: (**a**) 400 r/min; (**b**) 500 r/min; (**c**) 600 r/min; (**d**) 700 r/min.

Figure 17 shows the three-dimensional surface topography and the corresponding diagonal cross-sectional profiles of diamond specimens under different lapping speeds. It can be observed that, due to the extreme hardness of diamond, no obvious scratch defects are visible in the three-dimensional surface topography under WLI, regardless of the lapping speed, under the action of diamond abrasive grits. At a lapping speed of 400 r/min, due to the low speed, the number of contacts between abrasive grits and the specimen surface per unit time is relatively small, resulting in insufficient removal of surface defects with numerous pit defects remaining. The diagonal profile shows significant fluctuations with pronounced peaks and valleys, including five peaks higher than 0.04 μm and two valleys deeper than −0.04 μm. When the lapping speed reaches 600 r/min, the surface pit defects are significantly reduced, and the diagonal profile fluctuations are mitigated, with only one peak higher than 0.04 μm and one valley deeper than −0.04 μm, indicating that the interaction between abrasive grits and the workpiece is relatively stable at this speed, yielding the best surface quality. When the lapping speed reaches 700 r/min, the surface pit defects increase, and the diagonal profile fluctuations become more severe, with one sharp narrow valley exceeding −0.05 μm and one broad valley exceeding −0.04 μm.

### 5.2. Effect of Different Lapping Pressures on Fine Lapping Performance

**Figure 18:**
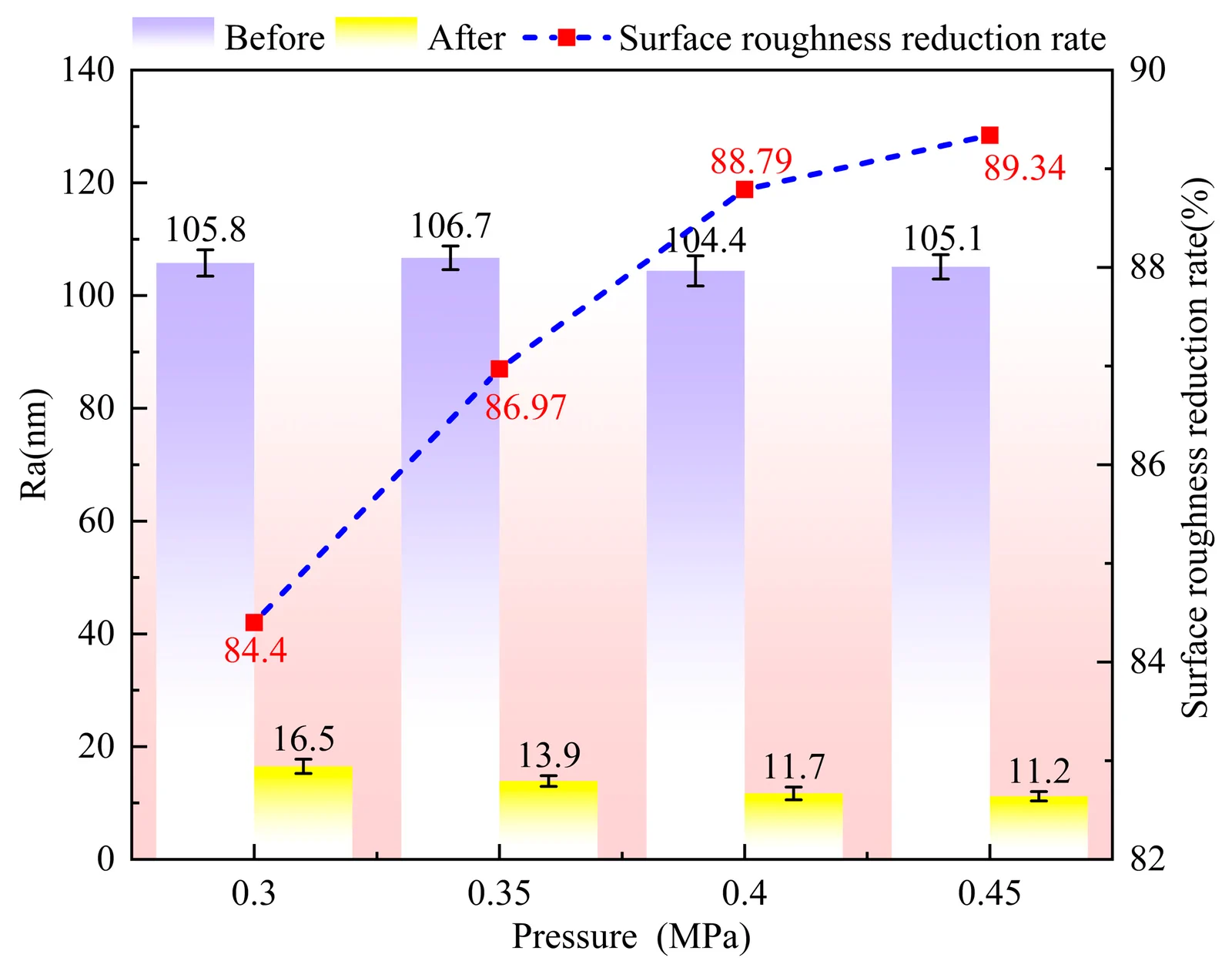
Surface roughness of samples before and after processing under various lapping pressures.

To systematically investigate the influence of different lapping pressures on fine lapping performance, four groups of lapping pressures—0.3 MPa, 0.35 MPa, 0.4 MPa, and 0.45 MPa—were selected for fine lapping experiments, with the lapping speed maintained at 600 r/min, a lapping duration of 12 h, and W3.5 abrasive grits. Figure 18 shows the surface roughness of the specimens before and after processing under different lapping pressures. The surface roughness Ra decreased with increasing lapping pressure. When the lapping pressure increased from 0.3 MPa to 0.45 MPa, the corresponding surface roughness values of the diamond specimens were 16.5 nm, 13.9 nm, 11.7 nm, and 11.2 nm, respectively, with a maximum difference of 5.3 nm. When the lapping pressure increased from 0.3 MPa to 0.4 MPa, the ploughing and scratching actions between the abrasive grits and the specimen surface were enhanced. This enhancement enabled more effective removal of surface micro-protrusions, pits, and other defects, resulting in a significant decrease in surface roughness Ra. When the lapping pressure was further increased to 0.45 MPa, the improvement in surface roughness diminished, with a reduction of only 0.5 nm, indicating that the improvement in surface quality by pressure increase tended to saturate. As the lapping pressure increased from 0.3 MPa to 0.45 MPa, the surface roughness improvement rates of the specimens were 84.40%, 86.97%, 88.79%, and 89.34%, respectively, showing a gradual increase. Therefore, appropriately increasing the lapping pressure within a certain range is beneficial to improving the lapped surface quality, but excessive lapping pressure yields limited additional improvement in surface quality.

**Figure 19:**
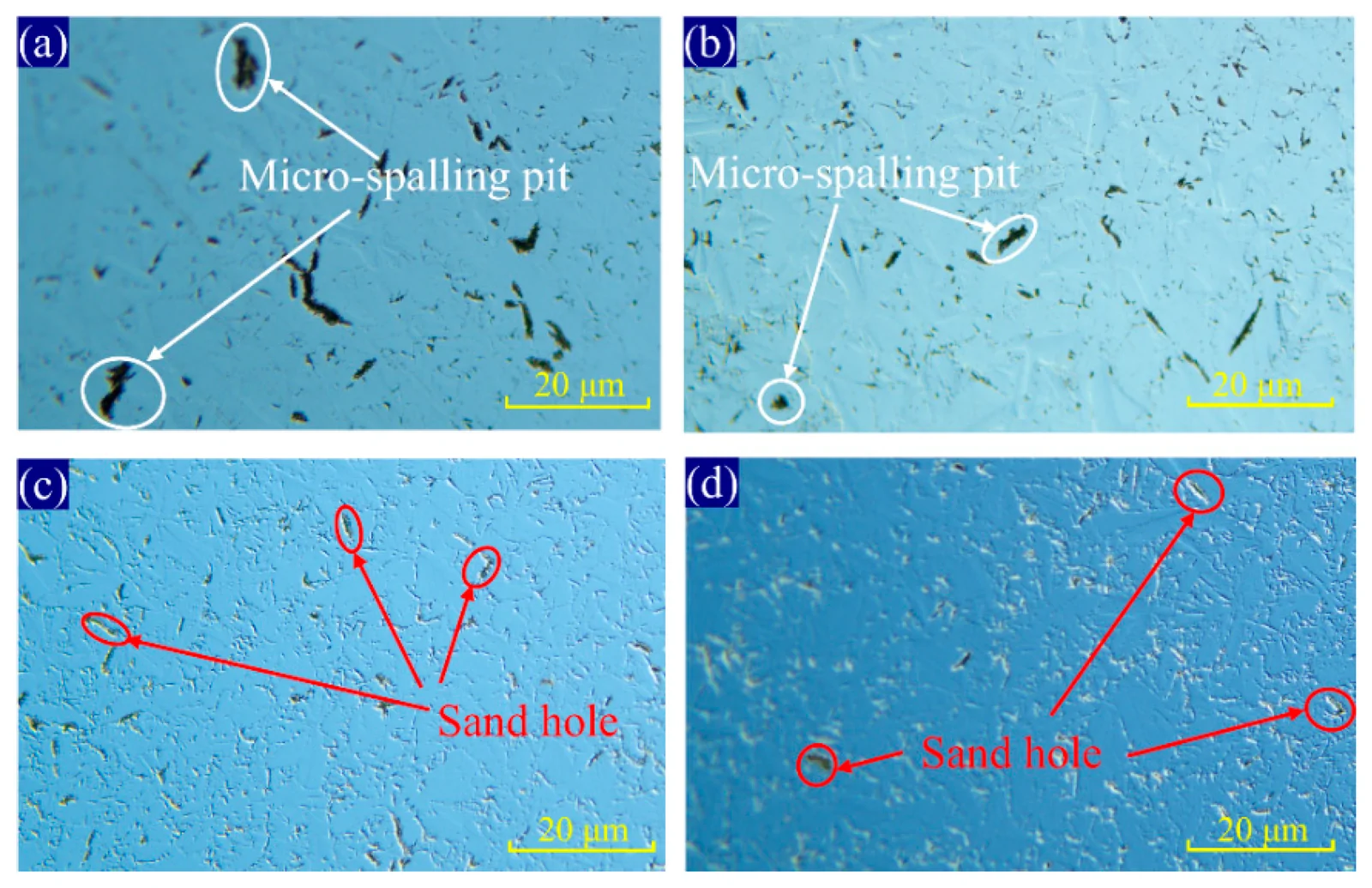
Optical micrographs under various lapping pressures: (**a**) 0.3 MPa; (**b**) 0.35 MPa; (**c**) 0.4 MPa; (**d**) 0.45 MPa.

Figure 19 shows the surface morphologies under different lapping pressures. Surface defects decreased with increasing lapping pressure. At a lapping pressure of 0.30 MPa, the mechanical action between the abrasive grits and the specimen was relatively weak, resulting in low cutting efficiency. As a consequence, the pit defects remaining from the rough lapping stage could not be effectively removed, and the overall surface quality of the specimen was poor. When the lapping pressure was increased to 0.4 MPa, the pit defects on the surface gradually became shallower, and the surface quality was significantly improved. When the lapping pressure was further increased to 0.45 MPa, most of the pit defects were removed, and the surface defects were predominantly small sand holes, achieving the best surface quality among the group.

**Figure 20:**
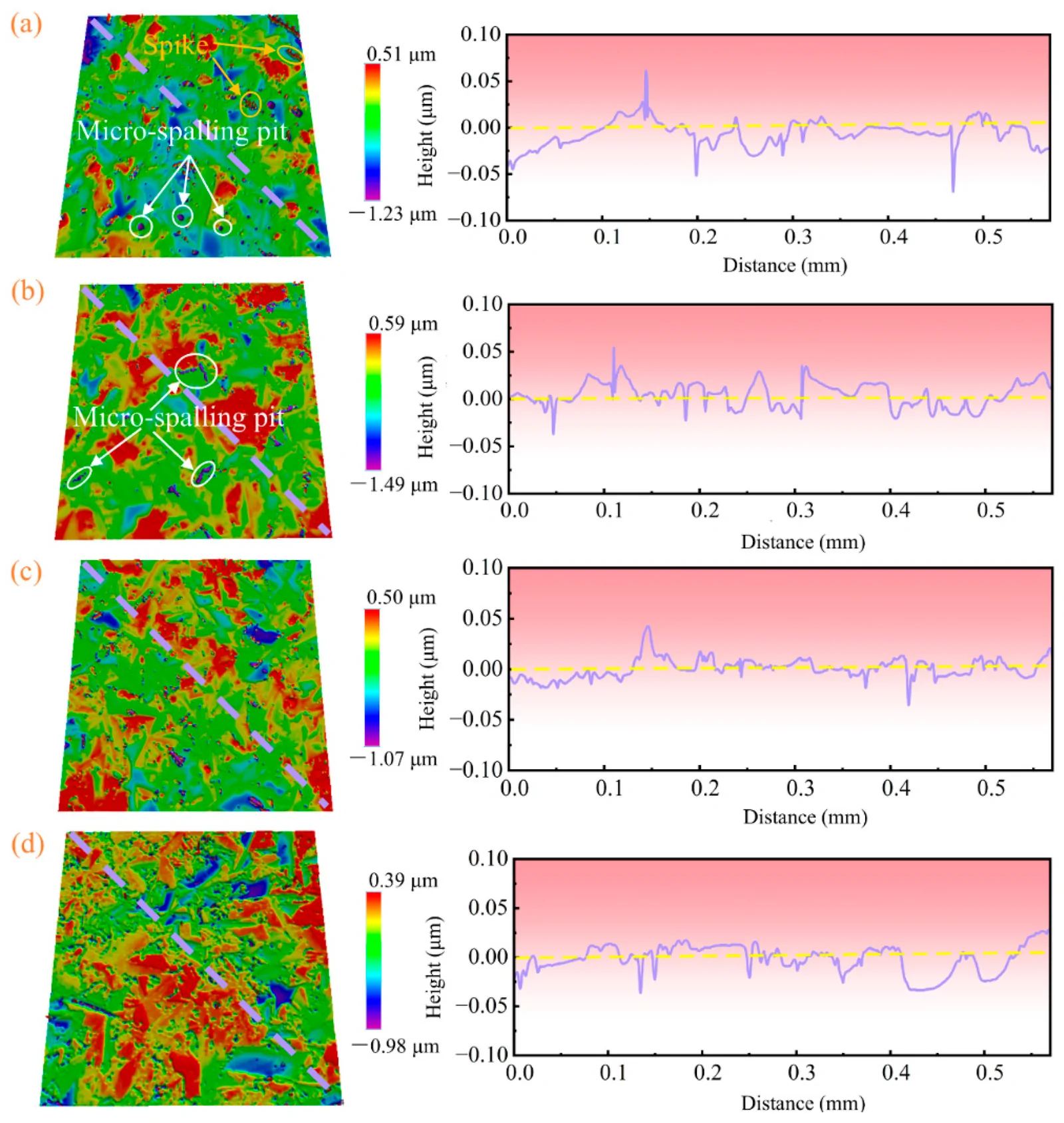
3D surface topography and diagonal profiles of diamond specimens under various lapping pressures: (**a**) 0.3 MPa; (**b**) 0.35 MPa; (**c**) 0.4 MPa; (**d**) 0.45 MPa.

Figure 20 shows the three-dimensional surface topographies and corresponding diagonal profile curves of diamond samples at different lapping pressures. When the lapping pressure was 0.3 MPa, due to the weak micro-cutting action of the abrasive particles, a large number of pits left from the rough lapping process and incompletely removed peak defects remained on the sample surface. The diagonal profile curve exhibited severe fluctuations, with three valleys deeper than −0.04 μm (two of which exceeded −0.05 μm) and one peak higher than 0.05 μm, indicating poor surface flatness. As the lapping pressure increased to 0.4 MPa, the micro-cutting action between the abrasive particles and the sample was enhanced, leading to more effective removal of surface micro-protrusions and pits. The diagonal profile curve gradually became smoother, and the surface flatness of the sample was significantly improved. When the lapping pressure reached 0.45 MPa, no obvious pit defects were observed on the sample surface, and the diagonal profile curve showed no distinct peaks higher than 0.04 μm or valleys deeper than −0.04 μm, indicating further improvement in surface quality.

## 6. Analysis of Material Removal Mechanism of PCD

The above parameter optimization experiments have clarified the influence of speed and pressure on surface roughness. However, experimental approaches alone are insufficient to reveal the underlying mechanisms at the atomic scale. The saturation effect of pressure on surface roughness improvement, as well as the differential contributions of pressure and speed to material removal and SSD, all involve atomic-scale interactions at the abrasive–workpiece interface, which cannot be directly observed by conventional characterization methods. To address this, an MD model for nanoscale lapping of PCD was constructed to elucidate, at the atomic scale, the influence mechanisms of pressure and velocity on removal behavior and SSD.

### 6.1. Model Construction and Simulation Parameter Settings

**Figure 21:**
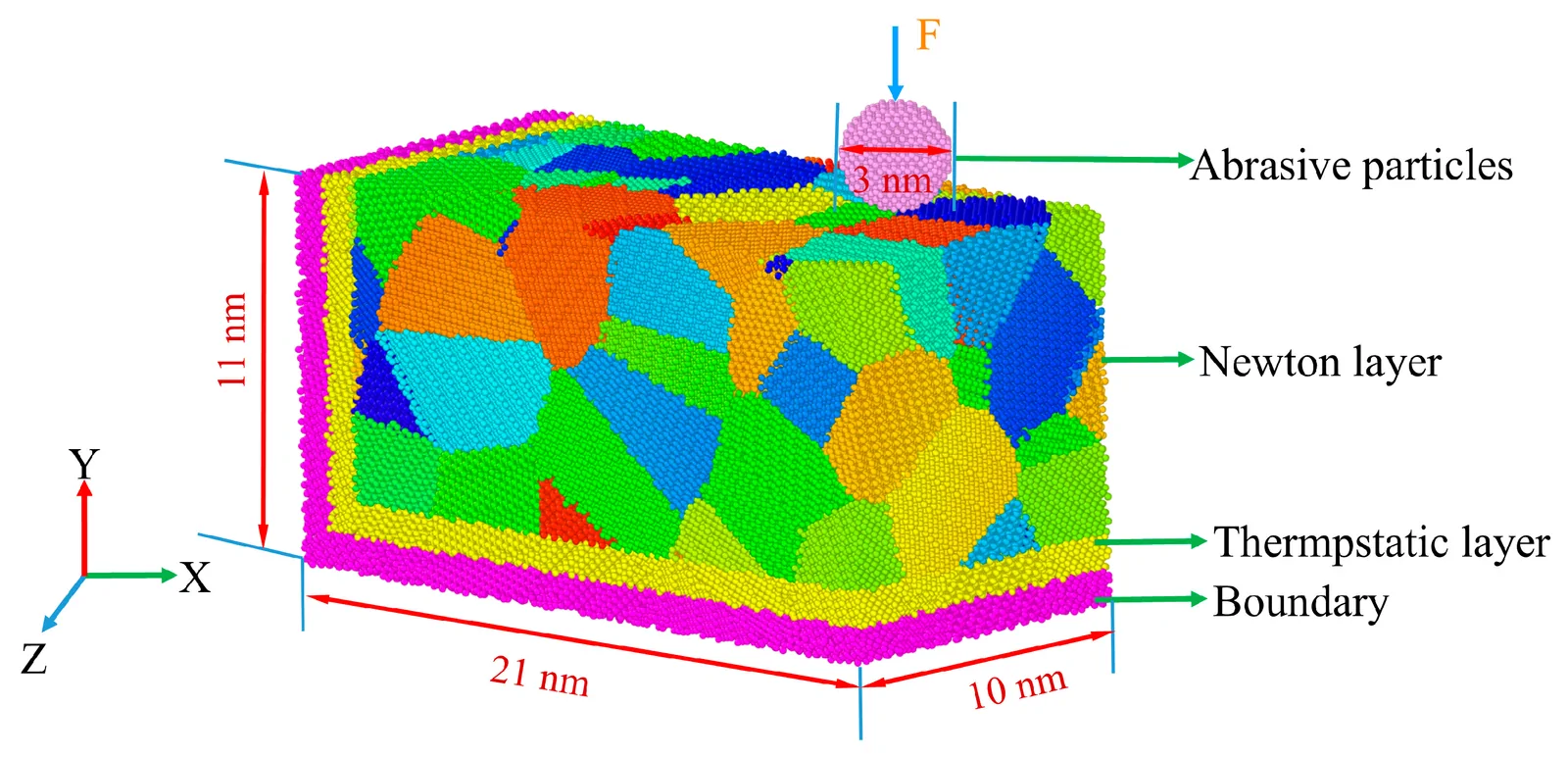
PCD lapping mode.

Figure 21 shows the diamond lapping model established in this simulation, with model dimensions of 10 nm × 11 nm × 21 nm. The model is divided into three layers: the boundary layer, the Newtonian layer, and the thermostat layer. To ensure model stability, the atoms in the boundary layer were fixed, and the temperature of the atoms in the thermostat layer was maintained at 300 K to ensure reasonable outward heat conduction during the lapping process. The atomic motion in the Newtonian layer follows Newton’s second law. In this simulation, the NVE ensemble (microcanonical ensemble, with constant Number of atoms, Volume, and total Energy) is adopted for the Newtonian layer to ensure strict energy conservation and avoid artificial thermal interference. The boundary conditions are set as p p p (periodic boundary conditions in all three directions), where periodic boundaries are applied along the x, y, and z axes to mimic an infinite bulk material. The Tersoff potential [20] was employed to describe the C–C interactions within the diamond workpiece and between the diamond abrasive and the workpiece. This potential has been widely used in simulating nanoindentation, grinding, and structural evolution of diamond [21,22,23]. The lapping abrasive was a single-crystal diamond abrasive with a diameter of 3 nm. The PCD lapping model was constructed using the Voronoi geometric method in Atomsk [24], by which the spatial region within a 10 nm × 11 nm × 21 nm box was randomly divided into a specified number of polyhedral grains according to the input number of grains. The specific simulation parameters are listed in Table 1. All simulations are performed using the Large-scale Atomic/Molecular Massively Parallel Simulator (LAMMPS) package. LAMMPS is a classical MD simulator that solves Newton’s equations of motion for atomic systems using a finite-difference time integration scheme (velocity-Verlet algorithm), enabling efficient and accurate simulation of nanoscale material removal processes [25].

### 6.2. Analysis of Crystal Structure Transformation and Surface Morphology Evolution Process

**Figure 22:**
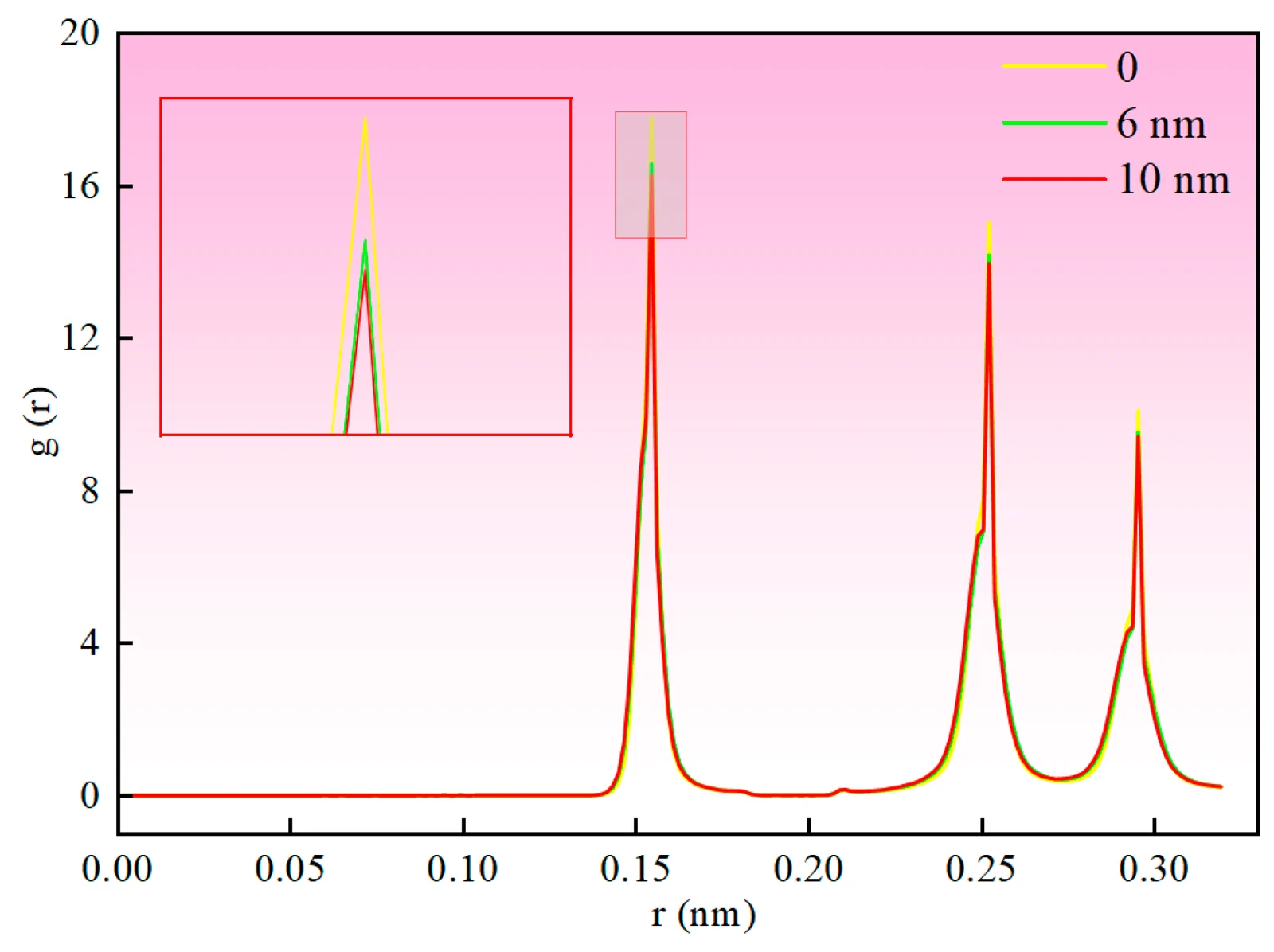
Radial distribution function at different machining distances.

The radial distribution function (RDF) is used to analyze the transformation of the workpiece crystal structure. This function is defined as the average probability of finding an atom around a central atom [26] and serves as an important indicator for characterizing crystal structure distribution. Figure 22 presents the radial distribution functions corresponding to different machining distances under conditions of a pressure of 800 nN and a rotational speed of 100 m/s. The results show distinct characteristic peaks at 0.154 nm, 0.25 nm, and 0.29 nm, exhibiting typical diamond crystal structure features. Among them, the first peak at 0.154 nm corresponds to the nearest neighboring C–C covalent bonds in the first coordination shell, directly characterizing the ordered structure of sp^3^ hybridized bonds. As the lapping distance increases, the intensity of this peak significantly weakens, indicating that the atomic order is gradually disrupted and the crystal begins to show a trend of amorphization.

**Figure 23:**
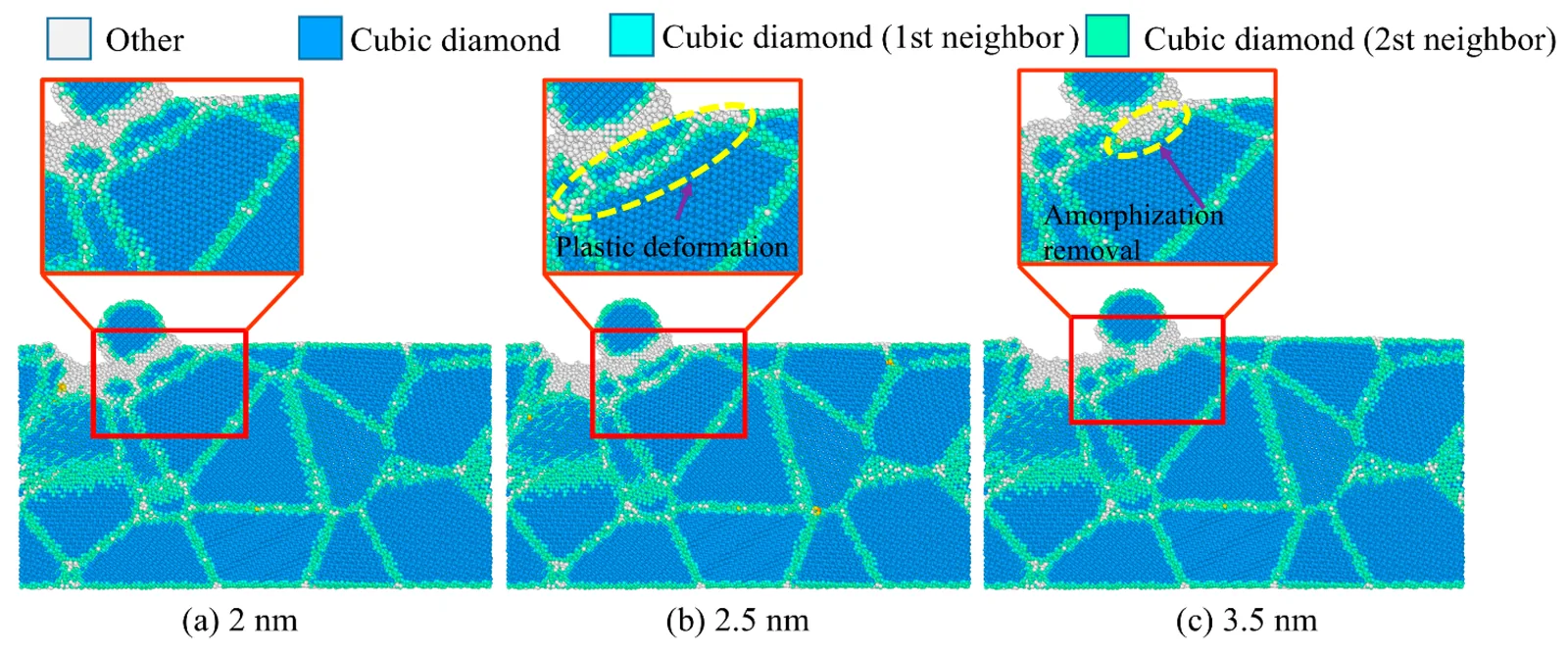
Material removal mechanism of PCD.

Figure 23 illustrates the material removal process of polycrystalline diamond during lapping. At the initial stage of abrasive contact, plastic deformation preferentially occurs in the grain boundary regions. This is because the atomic arrangement at grain boundaries is loose and the binding energy is relatively low, making them less resistant to external loading than the grain interiors. As the normal load increases, the atoms ahead of the abrasive grain are subjected to combined compression and shear, leading to intensified stress concentration at the grain boundaries. This causes atomic bond breakage and a decrease in lattice order, resulting in amorphization. As the abrasive grain continues to advance, the atoms in the amorphized region gradually detach from the matrix, forming wear debris, thereby achieving material removal.

**Figure 24:**
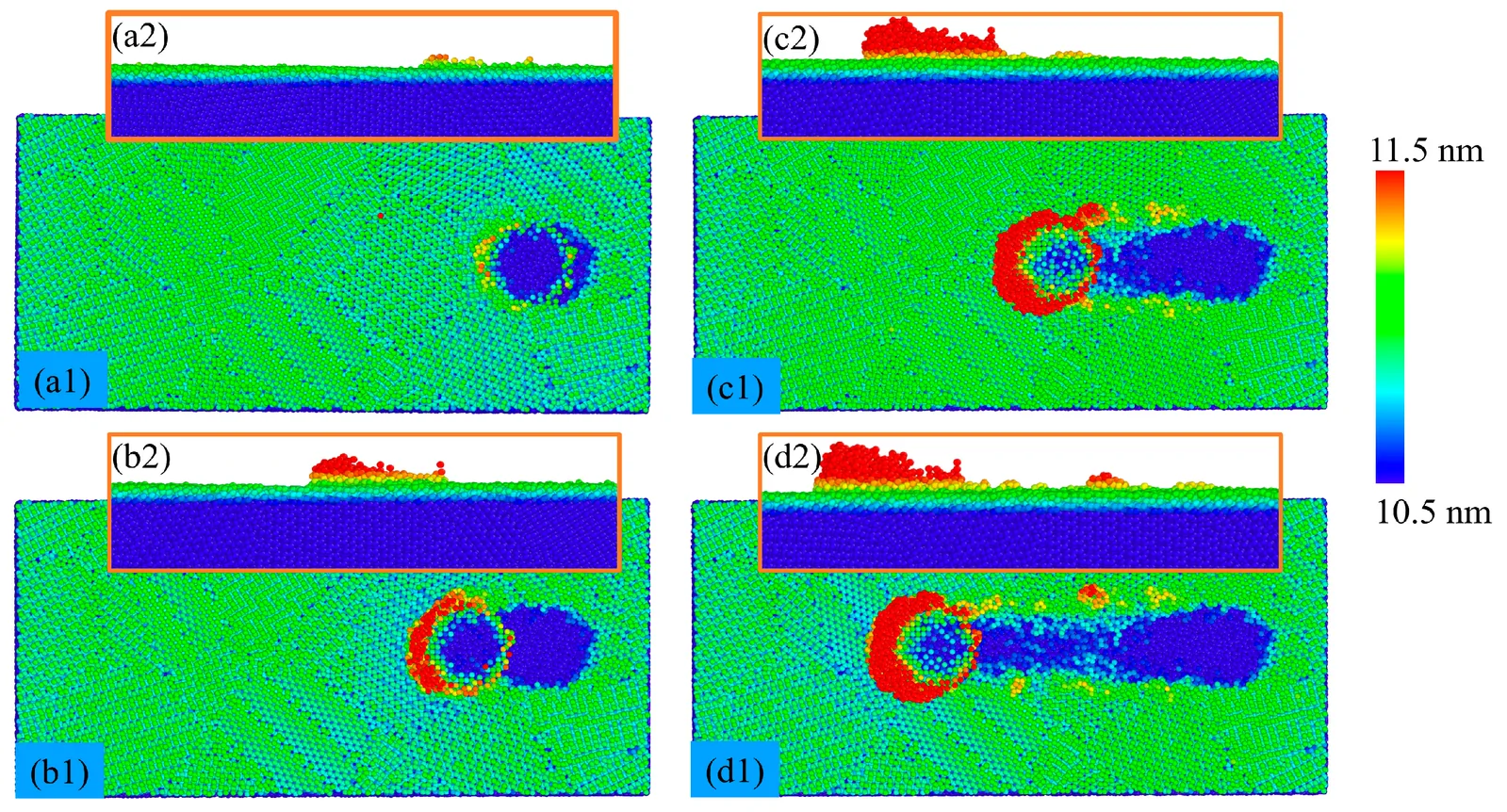
Atomic accumulation at different machining distances: (**a1**,**a2**) 1 nm, (**b1**,**b2**) 3 nm, (**c1**,**c2**) 6 nm, (**d1**,**d2**) 10 nm.

Figure 24 shows the surface morphologies obtained when a diamond abrasive grain slides across the surface of a PCD workpiece under a cutting pressure of 800 nN and a sliding speed of 100 m/s, at different sliding distances. To gain a deeper understanding of the surface formation mechanism during the nanoscale lapping process of PCD, color variations are used to represent the displacement of workpiece atoms in the Z-direction, where brighter colors indicate larger Z-direction displacements. Due to the plowing effect generated by the forward movement of the diamond abrasive grain, a large number of atoms are removed from the surface, resulting in the formation of two distinct regions. In Figure 24b–d, the atoms shown in red have been separated from the surface of the PCD substrate in the Z-direction and form nanoscale lapping chips in front of the abrasive grain. Meanwhile, accumulation phenomena occur on both sides of the groove and in front of the abrasive grain. As the nanoscale lapping process proceeds, the corresponding chip height and accumulation width gradually increase.

### 6.3. Analysis of the Effect of Applied Force on the Removal Behavior and SSD of PCD Substrate

**Figure 25:**
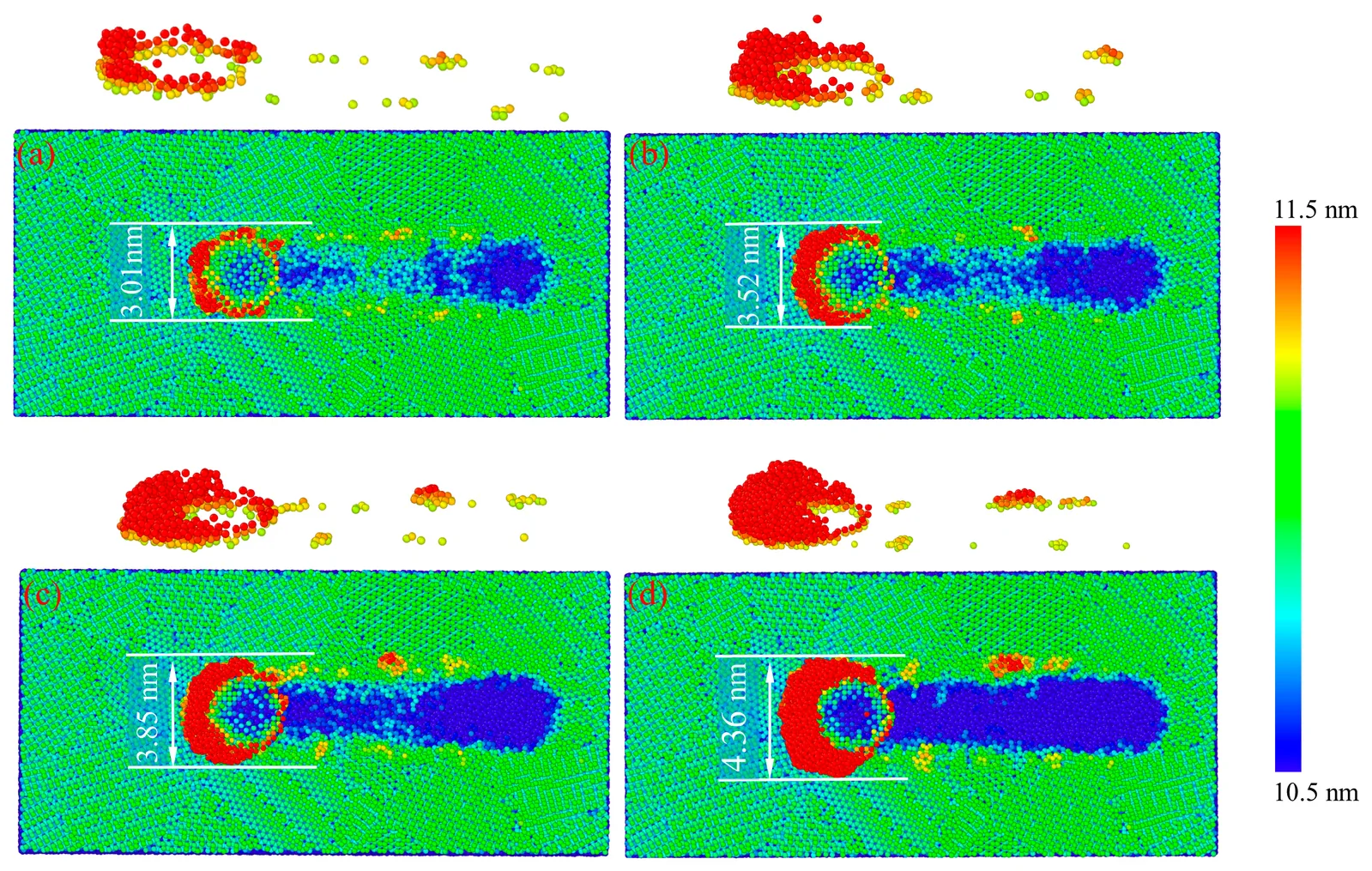
Surface topography of the PCD substrate after a machining distance of 10 nm under different applied forces: (**a**) 600 nN, (**b**) 700 nN, (**c**) 800 nN, (**d**) 900 nN.

**Figure 26:**
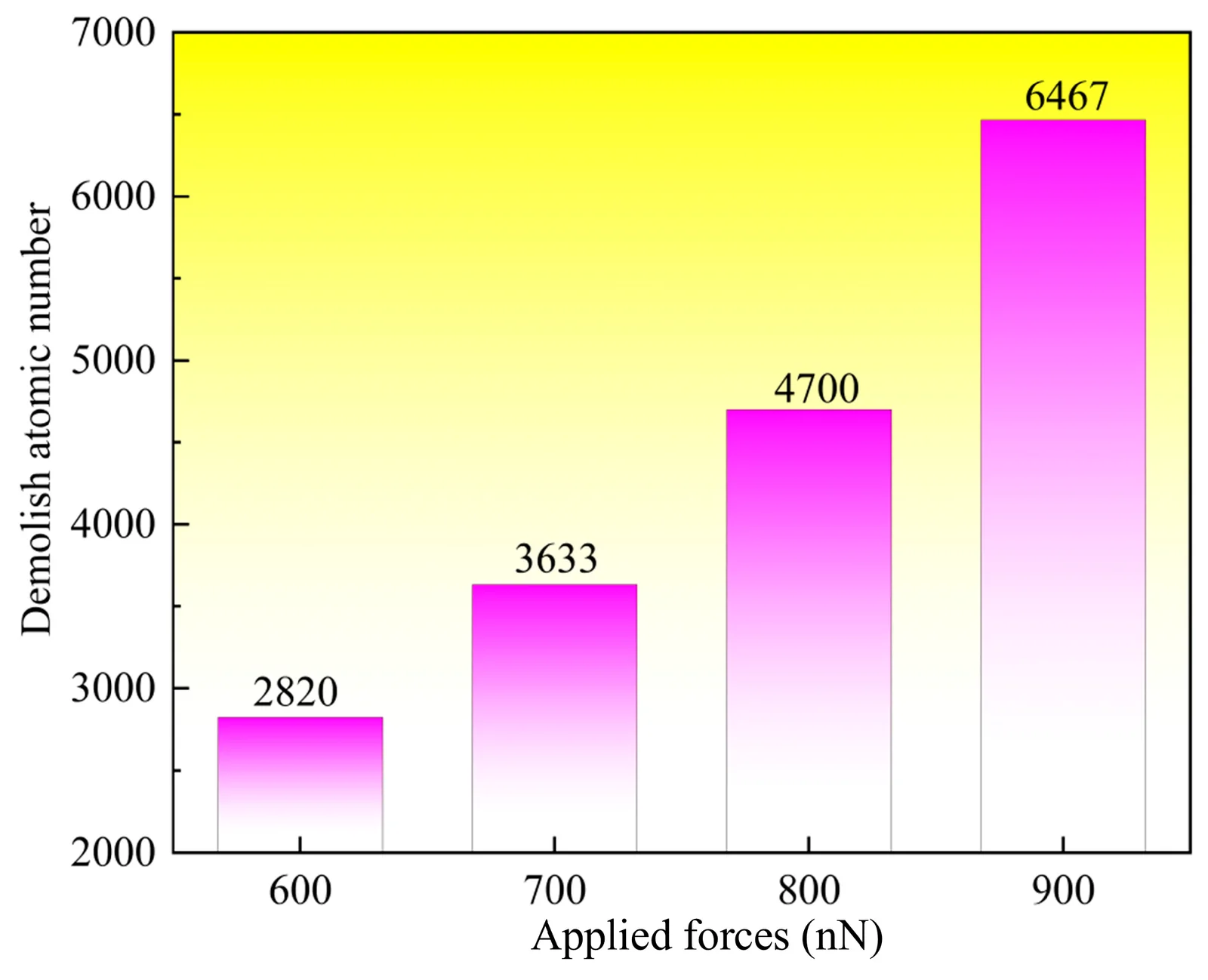
Number of atoms removed from a PCD substrate at different applied forces with a processing distance of 10 nm.

Figure 25 shows the surface topography of a PCD substrate after a machining distance of 10 nm under different normal loads using a single-crystal diamond abrasive grain. The width of the accumulated material on the machined workpiece surface increases with increasing indentation depth, and the groove width also becomes wider, exceeding the width of the diamond abrasive grain. Throughout the scratching process, a small amount of material accumulation forms on both sides of the abrasive grain at different indentation depths, with more material accumulation concentrated in front of the abrasive grain. The machined groove deepens with increasing pressure. As the normal load increases from 600 nN to 900 nN, the width and thickness of the surface accumulated atoms increase from 3.01 nm to 4.36 nm, representing an increase of 44.9%. Figure 26 shows the number of atoms removed from the PCD workpiece by a single-crystal diamond abrasive grain under different applied forces at a machining distance of 10 nm. The influence of pressure on the number of surface atoms removed is significant. As the lapping pressure increases from 600 nN to 900 nN, the number of removed surface atoms increases from 2820 to 6467, representing an increase of 129.3%.

**Figure 27:**
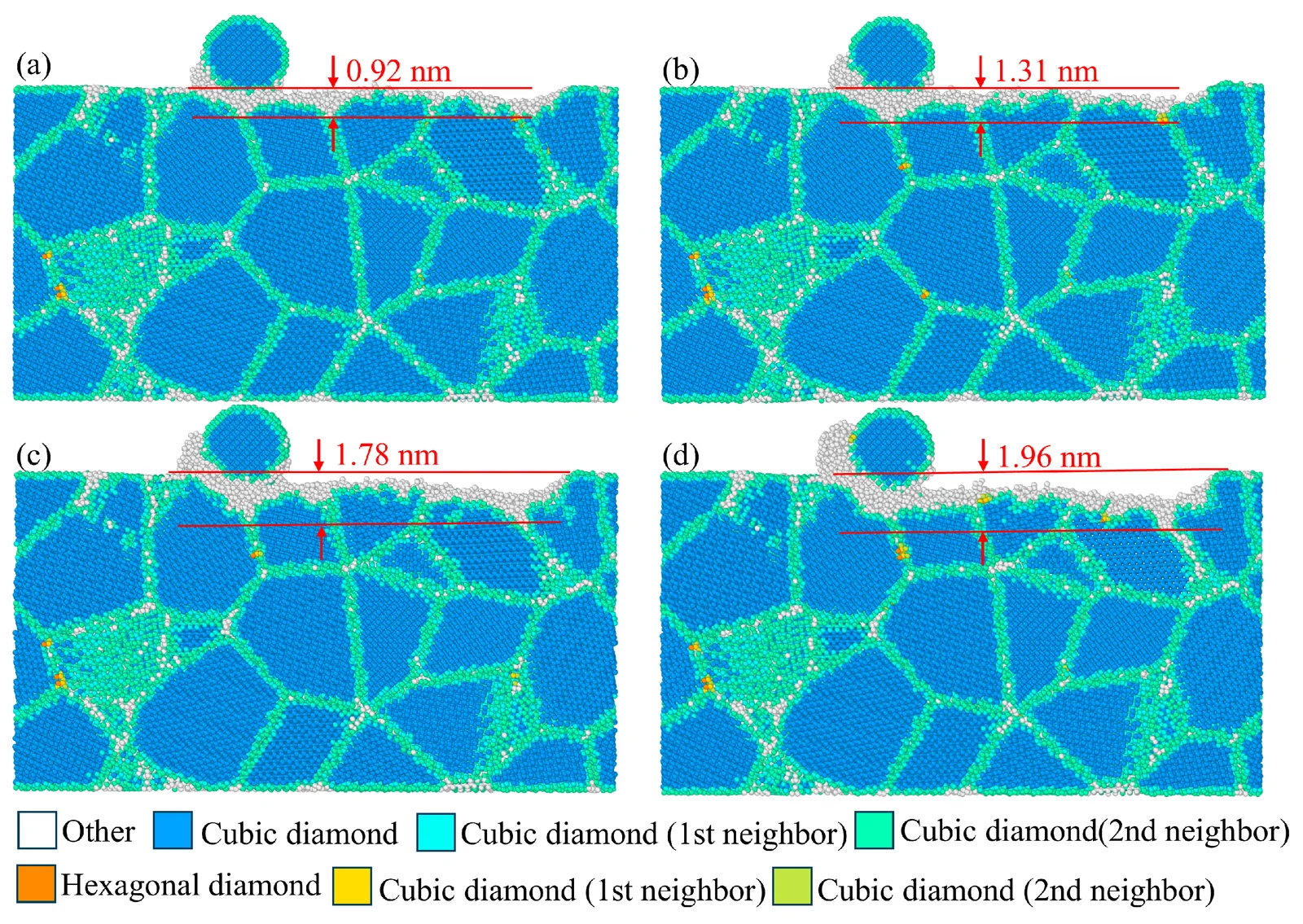
SSD under different applied forces: (**a**) 600 nN, (**b**) 700 nN, (**c**) 800 nN, (**d**) 900 nN.

Figure 27 shows the SSD morphology of a PCD workpiece generated by a single-crystal diamond abrasive grain under different normal loads at a machining distance of 10 nm. In Figure 27, the dark blue region represents atoms with a regular diamond structure, the white region represents atoms in the non-diamond phase, and the light blue and green regions represent a structurally regular transition layer between diamond-structured atoms and amorphized atoms. As the normal load gradually increases, the SSD layer thickness of the PCD substrate increases accordingly. This is because the increased normal load enhances the extrusion between the abrasive grain and the substrate surface atoms, causing the damage layer to extend deeper into the substrate and significantly deepening the SSD in the compressed region. The damage layer is not limited to the surface but further develops into the substrate interior, leading to the progressive accumulation of localized SSD. When the applied forces increases from 600 nN to 900 nN, the surface damage layer thickness increases from 0.92 nm to 1.96 nm, representing an increase of 113%.

**Figure 28:**
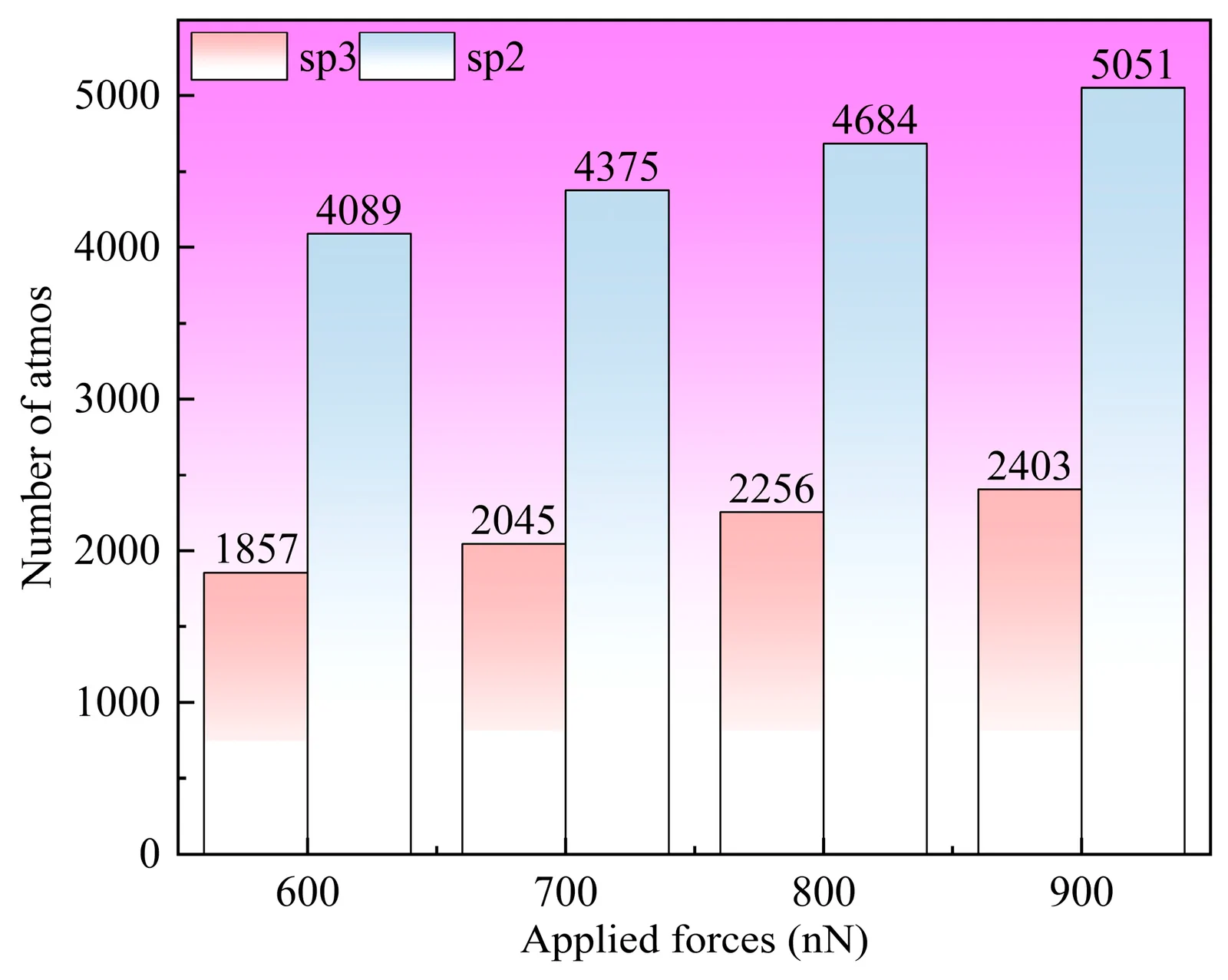
Number distribution of amorphous sp^3^ and sp^2^ atoms under various normal applied forces.

To further investigate the effect of pressure on the amorphization behavior, the amorphous atoms in the model were classified and counted. Figure 28 shows the quantitative distribution of two types of amorphous atoms under different normal applied forces. The results indicate that as the applied force increases from 600 nN to 900 nN, the number of amorphous sp^3^ atoms increases from 1857 to 2403, corresponding to an increase of 29.4%, while the number of amorphous sp^2^ atoms increases from 4089 to 5051, an increase of 23.5%. Both types exhibit significant growth with increasing applied force. This is primarily because the increase in normal applied force directly elevates both the contact compressive stress and shear stress between the abrasive grain and the workpiece. The increased compressive stress causes more atoms to undergo lattice compression distortion, where long-range order is disrupted while short-range tetrahedral coordination is preserved, forming amorphous sp^3^. The increased shear stress promotes interlayer sliding among more atoms, leading to the breakage of C–C bonds and subsequent re-bonding within the plane, forming amorphous sp^2^. As both stress components rise simultaneously with the increasing applied force, the two amorphous phases both increase correspondingly. In addition, the number of amorphous sp^2^ atoms is consistently approximately 2.2 times that of amorphous sp^3^ atoms, indicating that shear action dominates the amorphization process during abrasive scratching.

### 6.4. Analysis of the Effect of Velocity on the Removal Behavior and SSD of PCD Substrate

**Figure 29:**
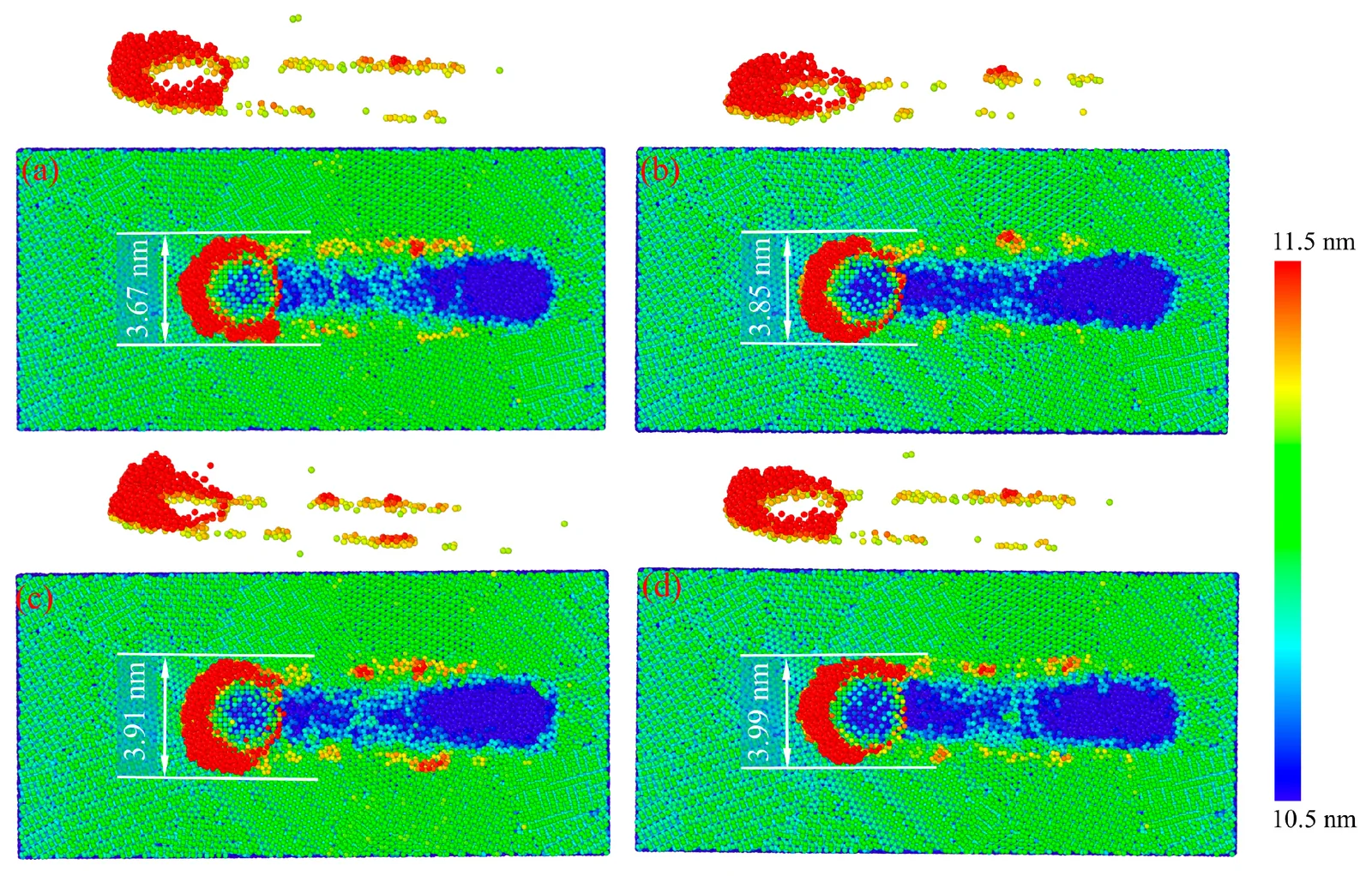
Surface topography of the PCD substrate after a machining distance of 10 nm under different sliding velocities: (**a**) 50 m/s, (**b**) 100 m/s, (**c**) 150 m/s, (**d**) 200 m/s.

**Figure 30:**
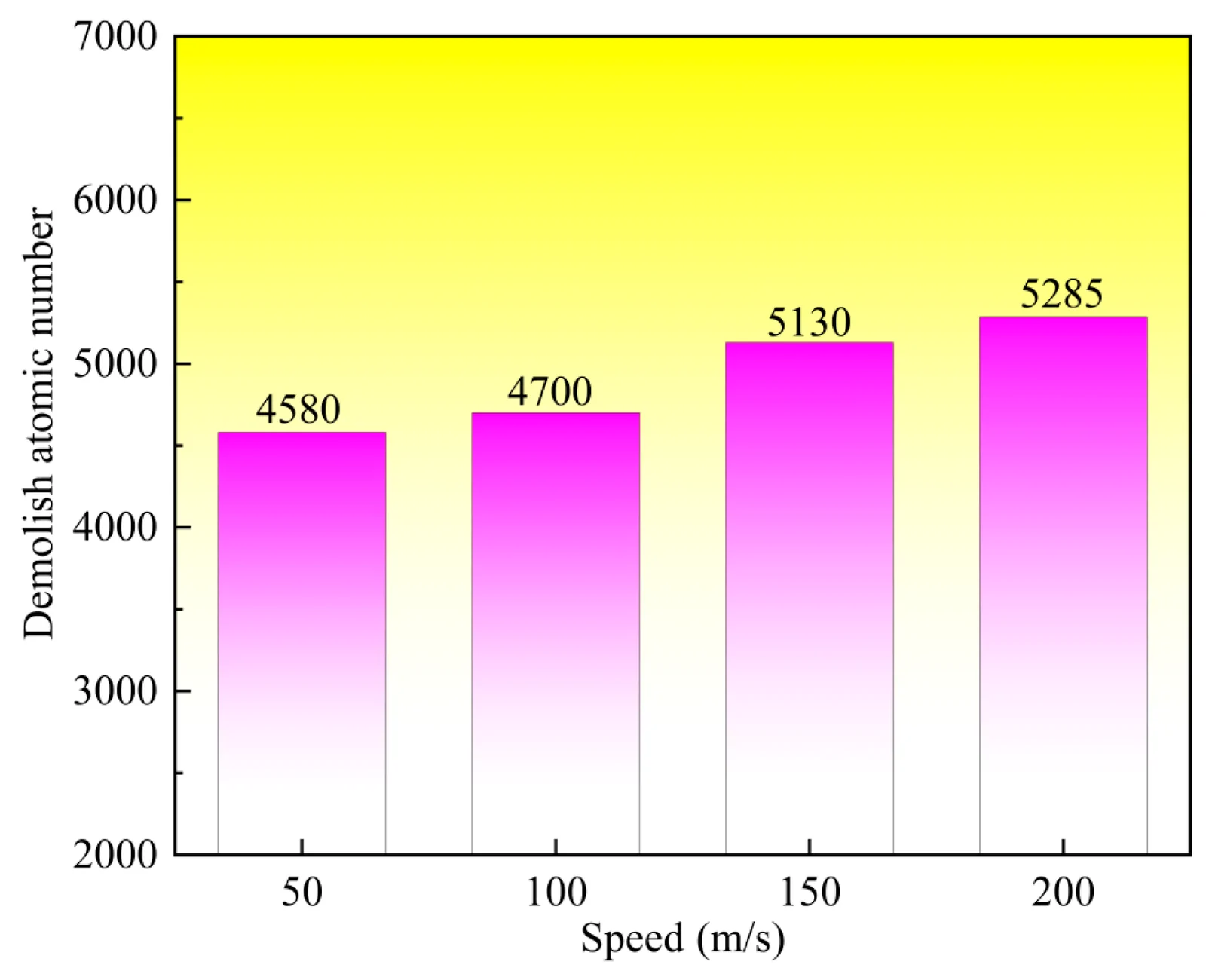
Number of atoms removed from a PCD substrate at different speeds with a processing distance of 10 nm.

Figure 29 shows the surface topography of a PCD workpiece generated by a single-crystal diamond abrasive grain under different velocities at a machining distance of 10 nm. At different velocities, the material accumulation formed by the abrasive grain is mainly concentrated in front of the grain, with only a small amount of accumulation on both sides. As the velocity increases, the surface accumulation in front of the abrasive grain does not increase significantly. When the lapping velocity increases from 50 m/s to 200 m/s, the width of surface accumulated atoms increases from 3.67 nm to 3.99 nm, representing an increase of 8.7%. Compared with the effect of pressure, the effect of velocity on the accumulation width is relatively small. Figure 30 shows the number of atoms removed from a PCD workpiece by a single-crystal diamond abrasive grain at different velocities with a machining distance of 10 nm. As the lapping velocity increases from 50 m/s to 200 m/s, the number of surface atoms removed increases from 4580 to 5285, representing an increase of only 15.4%. Compared with the significant effect of pressure on removal efficiency (129.3%), the effect of velocity on the total number of removed atoms within the range of 50–200 m/s is relatively limited, indicating that velocity is not the dominant factor in this nanoscale scratching process. Different from the macroscopic experimental results presented in Section 5.1, the effect of lapping speed on surface roughness is mainly governed by the effective contact probability of the abrasive grits under the action of centrifugal force. In contrast, in the MD simulations, a single abrasive grit always slides continuously with a constant indentation depth, without the centrifugal ejection or contact interruption that occurs in macroscopic processing. Under such ideal conditions, the material removal amount is primarily determined by the indentation depth of the abrasive grit, rather than by the sliding speed. Consequently, the sliding velocity exerts only a negligible influence on both the removal rate and the subsurface damage.

**Figure 31:**
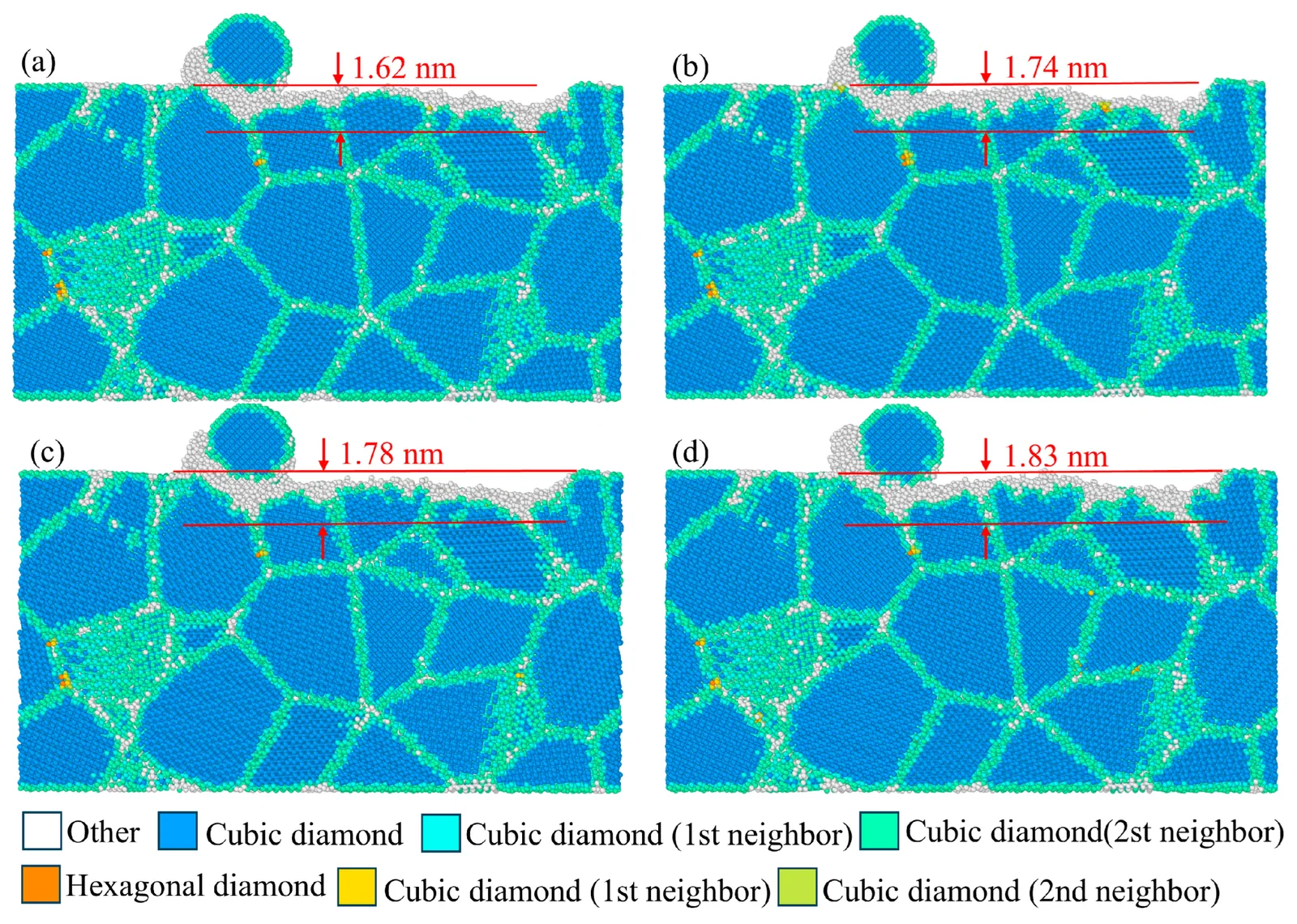
SSD of the PCD substrate after a machining distance of 10 nm under different sliding velocities: (**a**) 50 m/s, (**b**) 100 m/s, (**c**) 150 m/s, (**d**) 200 m/s.

Figure 31 shows the SSD morphology of a PCD workpiece generated by a single-crystal diamond abrasive grain at different velocities with a machining distance of 10 nm. It can be observed that the damage layer of the PCD is not limited to the surface but further develops into the substrate interior, leading to the progressive accumulation of localized SSD. As the lapping velocity increases from 50 m/s to 200 m/s, the surface damage layer thickness increases from 1.62 nm to 1.83 nm, representing an increase of 12.9%. Overall, the effect of velocity on SSD is relatively small.

**Figure 32:**
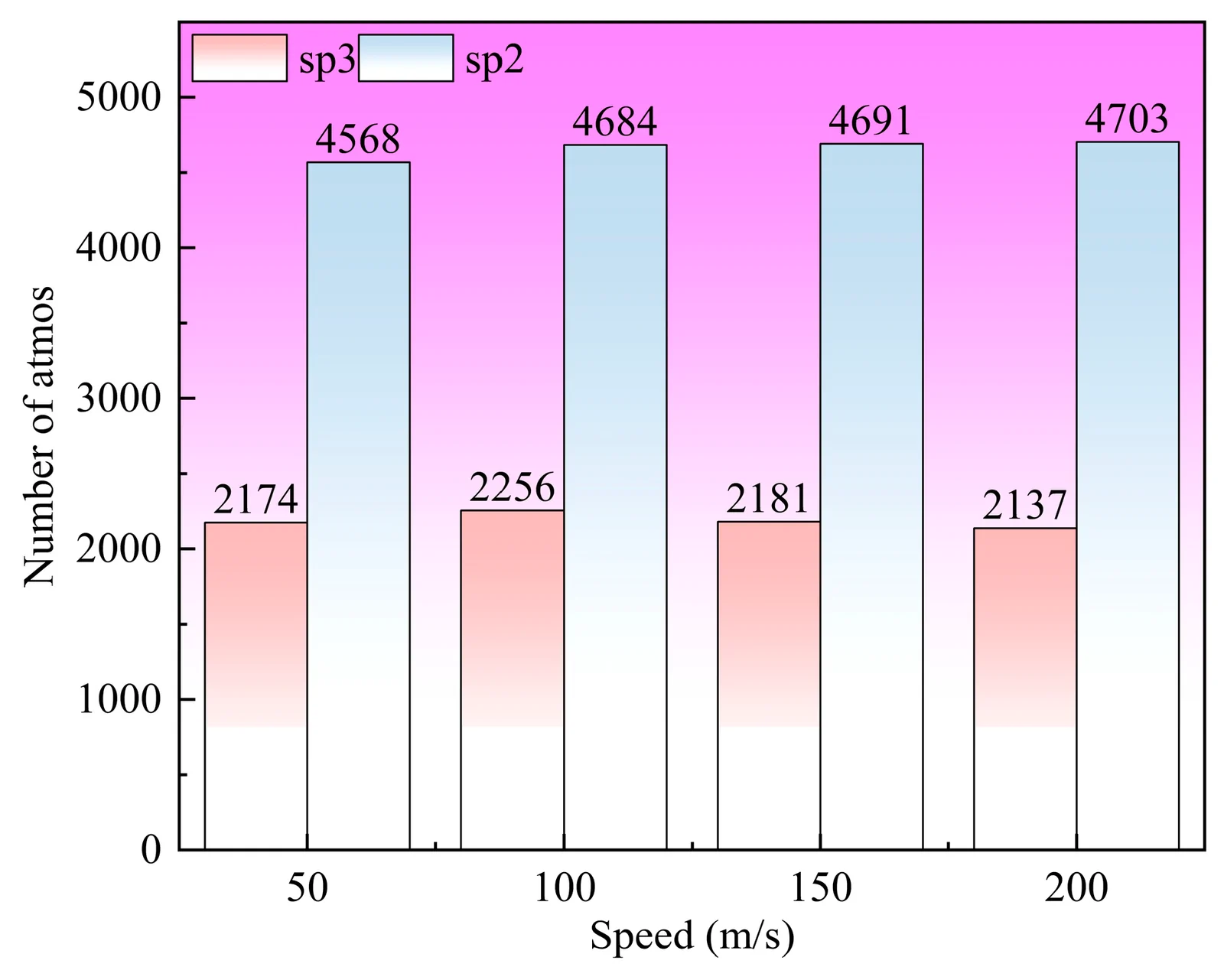
Quantitative distribution of amorphous sp^3^ and sp^2^ atoms at different scratching speeds.

The same classification and counting of amorphous atoms were performed under different velocity conditions. Figure 32 shows the quantitative distribution of the two types of amorphous atoms at various scratching speeds. As can be seen from the figure, within the range of 50–200 m/s, the number of amorphous sp^3^ atoms only increases from 4568 to 4703, with an increase of merely about 3.0%, while the number of amorphous sp^2^ atoms fluctuates slightly within the range of 2137–2256. The total number of amorphous atoms remains essentially stable. Compared with the significant changes observed under varying applied forces, the driving effect of speed on amorphization is rather limited. This is primarily because variations in speed do not alter the stress state of a single contact between the abrasive grain and the workpiece, but only affect the contact frequency and contact duration. The formation of amorphous sp^2^ not only requires bond breakage but also relies on the structural rearrangement of atoms within the plane, a process that requires a finite amount of time. When the speed is too high, some of the broken bonds are carried away by the abrasive grain before they can be rearranged.

## 7. Conclusions and Future Perspectives

This study adopted a “rough lapping + fine lapping” two-stage strategy to systematically investigate abrasive tool selection, process optimization, and MD simulations for mechanical lapping of polycrystalline diamond (PCD). The main conclusions are as follows: (1) In the rough lapping stage, the resin-bonded disc, characterized by low elastic modulus and moderate holding force that enable timely self-sharpening of abrasive grits, exhibited the best performance, reducing surface roughness Ra from 420 nm to 106 nm (a reduction of 74.76%). (2) In the fine lapping stage, the high-stiffness WC-Co disc achieved the best surface quality, with the smallest Raman FWHM of 5.2 cm^−1^, indicating the highest surface crystal ordering. (3) The effect of rotational speed on surface roughness follows a trend of first decreasing and then increasing, with the lowest Ra value obtained at 600 r/min. Regarding lapping pressure, when the pressure exceeds 0.4 MPa, a further increase in pressure yields a significantly diminished improvement in surface roughness, indicating a saturation effect. (4) MD simulations reveal that when the normal load increases from 600 nN to 900 nN, the number of removed atoms increases by 129.3%, but the SSD layer thickens by 113%. In contrast, within the speed range of 50–200 m/s, the removal rate increases by only 15.4% and the damage layer thickness by 12.9%, indicating a negligible effect. Therefore, pressure is the key parameter governing the trade-off between removal efficiency and surface integrity.

Future work will focus on three main directions. First, the lapping performance of PCD with different grain sizes will be investigated to understand the influence of microstructure on material removal behavior. Second, the synergistic effects of ultrasonic vibration assistance on the current lapping setup will be explored to further improve surface quality and processing efficiency. Third, the optimized process parameters obtained in this study will be scaled up for industrial application trials to verify their feasibility in large-scale production.

## Figures and Tables

**Table 1:** Simulation parameters.

Parameters	Simulated Values
Abrasive grain radius	1.5 nm
Workpiece dimensions	21 × 10 × 11 nm
Time step	1 fs
Boundary conditions	p p p
Ensemble settings	nve
Initial temperature	300 K
Potential function	Tersoff

## Data Availability

The original contributions presented in this study are included in the article. Further inquiries can be directed to the corresponding author.

## Abbreviations

The following abbreviations are used in this manuscript:

PCD	Polycrystalline diamond
MD	Molecular dynamics
SSD	Subsurface damage
